# Edible Aquatic Insects: Diversities, Nutrition, and Safety

**DOI:** 10.3390/foods10123033

**Published:** 2021-12-06

**Authors:** Min Zhao, Cheng-Ye Wang, Long Sun, Zhao He, Pan-Li Yang, Huai-Jian Liao, Ying Feng

**Affiliations:** 1 Key Laboratory of Breeding and Utilization of Resource Insects of National Forestry and Grassland Administration, Research Institute of Resource Insects, Chinese Academy of Forestry, Kunming 650224, China; mzhao@caf.ac.cn (M.Z.); cywang11@126.com (C.-Y.W.); sunlong1818@126.com (L.S.); hezhao@caf.ac.cn (Z.H.); yangpl1845@163.com (P.-L.Y.); 2 Institute of Leisure Agriculture, Jiangsu Academy of Agricultural Sciences, Nanjing 210014, China

**Keywords:** entomophagy, terrestrial insects, health benefits, contaminant, selenium, insect farming

## Abstract

Edible insects have great potential to be human food; among them, aquatic insects have unique characteristics and deserve special attention. Before consuming these insects, the nutrition and food safety should always be considered. In this review, we summarized the species diversity, nutrition composition, and food safety of edible aquatic insects, and also compared their distinguished characteristics with those of terrestrial insects. Generally, in contrast with the role of plant feeders that most terrestrial edible insect species play, most aquatic edible insects are carnivorous animals. Besides the differences in physiology and metabolism, there are differences in fat, fatty acid, limiting/flavor amino acid, and mineral element contents between terrestrial and aquatic insects. Furthermore, heavy metal, pesticide residue, and uric acid composition, concerning food safety, are also discussed. Combined with the nutritional characteristics of aquatic insects, it is not recommended to eat the wild resources on a large scale. For the aquatic insects with large consumption, it is better to realize the standardized cultivation before they can be safely eaten.

## 1. Introduction

Human beings have a very long and rich history of entomophagy, especially in Africa, Asia, and Latin America [1,2,3,4]. There are plentiful and colorful customs and cultures of entomophagy in these areas. To some ethnic groups, such as Mazahua and Nahuas in Mexico [5], Gelao in China [2], and Kiriwinians in Papua New Guinea [6], entomophagy not only means an indigenous practice of the consumption of insects, but is also imbibed into the ethnic culture and traditional ecological knowledge [7,8]. Entomophagy has decreased significantly with the development of modern agriculture; however, it still exists and plays important roles in the lives of people in underdeveloped areas. In recent years, considering their outstanding source of nutrition [9], low levels of greenhouse gas emissions [10], limited agricultural land being required [11], and potential socio-economic benefits, edible insects have been considered as valuable and sustainable alternative nutrition sources for food security [12]. The topics of “edible insects as food candidates” or “insects as an alternative protein source” have received a huge amount of attention [3,13]. Up until now, great progress in those topics has been achieved due to the encouragement of the greater use of insects in our diets by the Food and Agriculture Organization of the United Nations [14], as well as technological advances in research on the nutrition, safety and farming of insects.

Aquatic insects are composed of around 76,000 species, found in a wide range of aquatic (and semiaquatic) habitats, from springs, ponds and lakes to large rivers [15,16]. As edible insects, edible aquatic insects have a long history of utilization [5] too. These insects could have great potential to service humans with nutrition and health benefits; for example, the captured biomass of aquatic insects in Zaire (Central Africa) has already reached a very high value, with 16 tons/year in 1989 [17]. However, as compared to edible terrestrial insects, such as the black soldier fly, mealworm, cricket, and grasshopper, fewer aquatic insects appear in the market and daily life, and the same situation exists in terms of research. Insects inhabit a large variety of environments with different feeding habits; the majority of terrestrial insects are phytophagous, while the majority of aquatic insects are carnivores [18]. What is the difference or potentiality between the use of edible aquatic insects and terrestrial insects? In this review, the characteristics of aquatic insects were summarized to provide information to better develop and utilize this kind of resource. Here, ‘aquatic insects’ refers to aquatic and semi-aquatic insects in or from freshwater.

## 2. Aquatic Insects and Its Resource as Food and Feed

Aquatic insects have very rich species diversity, though aquatic insects represent only 10% of the insect species and only include 12 orders [19,20], and they share some of the same orders with terrestrial insects taxonomically. The relevant biology, natural habitats, and comparisons of aquatic insect orders have been well summarized by D. Dudley Williams and Siân S. Williams [19]. Six of the 12 orders of aquatic insects are likely to contain candidate species for food and feed [19,21,22]. They are Coleoptera (beetles), Diptera (true flies), Ephemeroptera (mayflies), Hemiptera (true bugs), Odonata (dragonflies/damselflies), and Trichoptera (caddisflies). Judging from the research and utilization reports from Southwest China and Japan, the order Megaloptera has the potential to be a candidate species, because it has been used as food and folk medicine for a long time, with remarkable economic value [23,24]. The insects are widely distributed all around the world, and its breeding technology of some species has gradually matured [25,26].

To update the list of edible aquatic insects, the methods of Macadam and Stockan [22] have been followed. Two main sources, Jongema (2017) [27] and Mitsuhashi (2016) [28], and other sources (Supplementary Table S1), were screened for the list. The list contains 329 species belonging to 153 genera, and 51 families coming from 46 countries (Table 1). Given that over 2000 insect species are eaten, edible aquatic insects account for about 15% of the total number. The species belong to eight orders, namely, Coleoptera, Odonata, Hemiptera, Diptera, Trichoptera, Megaloptera, Ephemeroptera, and Plecoptera, ordered from high to low. Among them, Coleoptera, Odonata, and Hemiptera contribute over 3/4 of the number of species, and they are all predatory. The naiad/larva is the main edible stage of aquatic insects. Most species are consumed in Mexico, Japan, China, Thailand, India, and Venezuela.

Noticeably, the number of species of edible aquatic insects might be underestimated [22] because the mainly edible stage of aquatic insects—naiads/larva—is morphologically undistinguishable most of the time. More and more species of edible aquatic insects will be identified with the help of molecular biology techniques [29].

## 3. Nutritional and Health Benefits of Edible Aquatic Insects

### 3.1. Protein Content and Amino Acid Composition of Aquatic Insects

Aquatic insects have an average protein content of 59.55% (Table 2), and this value is higher than that of conventional animal meats [30,31,32]. Furthermore, investigations identified that proteins from aquatic insects not only contain 45.93–62.01% essential amino acids (Table 2), but also have a good balance of different kinds of amino acids [33,34]. Meanwhile, the ratio of essential amino acids in aquatic insect proteins is close to human proteins, indicating a high nutritional value of aquatic insects [35].

Different edible insects have very different amino acid patterns [14,36,37]; we summarized the first limiting amino acid and highest amino acids in aquatic insects and compared these to terrestrial insects (Table 2). In aquatic insects, the average essential amino acid content is 51.60%, and the most abundant essential amino acid is Leu (7.8% on average). The limiting amino acids were Met + Cys (2.3% on average) and Trp (1.2% on average). The content of Glu (11.30% on average) is the highest among all the amino acids in aquatic insects. Jiang et al. compared the composition of the essential amino acids of dragonfly larvae in different regions and found that the difference is little, even between different species [38].

More attention has been paid to the amino acid content of terrestrial insects (Table 2) [32,37]. In terrestrial insects, the average essential amino acid content is 49.49%, and the most abundant essential amino acid is Leu (7.35% on average). The limiting amino acids were Thr (4.20% on average) and Trp (3.61% on average). The content of Glu (15.59% on average) is the highest among all the amino acids in terrestrial insects.

Although some studies have shown that the contents of some amino acids are different between aquatic and terrestrial insects, such as tryptophan [39], the difference is not significant judging from the average data, and this indicates that aquatic insects and terrestrial insects have similar amino acid compositions (Table 2). For example, Lys is present in both terrestrial edible insects and aquatic edible insects, with a rich content [38]. However, this essential amino acid is usually lacking in cereal protein, so both aquatic insects and terrestrial insects complement cereal protein in nutrition.

### 3.2. Characteristics of Fatty Acids in Aquatic Insects

As the second most abundant nutritional ingredient (only behind the protein content), fatty acids always play a crucial role in the growth and development of insects [40]. It is strongly believed that aquatic insects are also a rich source of saturated fatty acids (SFAs), monounsaturated fatty acids (MUFAs), and polyunsaturated fatty acids (PUFAs), especially PUFAs, such as linoleic acid (18:2), linolenic acid (18:3), arachidonic acid (20:4, AA), and eicosapentaenoic acid (20:5, EPA) [41,42]. In general, linoleic acid, linolenic acid, AA, and EPA belong to the omega-6 or omega-3 fatty acid family, which are beneficial to the health of human beings and must be obtained from the diet [43]. Table 3 presents some fatty acid compositions of selected aquatic and terrestrial insects. Noticeably, much research is carried out on the fatty acid composition of edible terrestrial insects. In contrast, there are much fewer data of the fatty acid composition of edible aquatic insects. For edible aquatic insects, SFAs are dominated by palmitic acid (16:0) and, to a lesser extent, by stearic acid (18:0), and oleic acid (18:1) is the most abundant composition among MUFAs, which is generally similar to the reported terrestrial species. In addition, edible terrestrial insects have a higher content of linoleic acid (18:2), with the exception of a few species, in comparison to aquatic groups. The great difference between aquatic insects and terrestrial insects is that the former are significantly enriched in PUFAs that contain four or more double binds, mainly in terms of AA and EPA [44,45,46]. The content of unsaturated fatty acids (UFA), palmitic acid, and oleic acid in the oil of dragonfly naiads is high, and the oil contains 1.23–7.05% odd carbon fatty acids (OCFA), which have the characteristics of general insect oil. Meanwhile, the oil of dragonflies contains some long-chain polyunsaturated fatty acids, including AA, EPA, and docosahexaenoic acids (DHA), which is similar to freshwater fish, and the similarity might result from the aquatic life stage of dragonfly naiads [47]. The presence of relatively substantial amounts of these PUFAs was possibly associated with membrane fluidity, because PUFAs have a lower melting point than SFAs or MUFAs, which helps aquatic insects to better adapt to the cold-water environment. When these aquatic insects were transferred to land and became adults, they were expected to have less need for cold endurance, and could be found to have low levels of ARA and EPA, or even undetectable levels [48].

### 3.3. Characteristics of Mineral Elements in Aquatic Insects

Minerals are particularly rich in insect-based foods [49,50], which indicate that insect-based foods are excellent mineral providers [51]; for example, both aquatic and terrestrial insects have higher contents of calcium, iron, and zinc compared with common meat [32].

When edible insects are divided into aquatic insects and terrestrial insects, the difference in their mineral element contents shows great heterogeneity in different studies. Sometimes, aquatic and terrestrial insects have similar elemental compositions [52]; for example, they have almost the same concentration of zinc [30,53]. However, for calcium and iron contents, studies have shown that there is a considerable gap between aquatic and terrestrial insects. A much higher calcium content was identified in aquatic insects (24.3–96 mg/100 g) [30] than in different terrestrial insects (0.0012–0.126 mg/100 g) [54]. The iron contents in aquatic insects (e.g., *Lethocerus indicus* 410 mg/100 g, *Hydrophilus olivaceous* 461 mg/100 g) [30] are significantly higher than in terrestrial insects (*Bombyx mori* 1.8 mg/100 g [55], *Cirina forda* 5.34 mg/100 g) [56].

Dragonflies are usually consumed as a dish in Southwest China, and their selenium content has been a particular concern [57]. In particular, the selenium content of common edible insects in China has been analyzed, and it was found that the average selenium content of terrestrial insects was 0.15 mg/kg, and that of aquatic insects was 0.31 mg/kg, which was two-fold higher than that in terrestrial insects [58].

To better compare the mineral element contents of aquatic and terrestrial insects as a whole, we summarized the insect species with relatively complete data, and divided them into aquatic and terrestrial insects. The comparisons of the average mineral element content between aquatic and terrestrial insects are obviously different from the comparison results from several separate literatures. In general, the contents of iron and sodium in aquatic insects were significantly higher than those in terrestrial insects, while the contents of magnesium and potassium were significantly lower than those in terrestrial insects (Table 4).

**Table 2:** Amino acid composition of edible aquatic insects and selected edible terrestrial insects.

Order	Species	Edvelopmental Stage	Protein (%)	Amino Acid Composition (% of Total Amino Acids or Protein)																		Total Amino Acids (g/100 g DM)	Reference
				Val	Ile	Leu	Lys	Tyr	Thr	Phe	Trp	His	Met + Cys	Total EAA ^++^	Arg	Asp	Ser	Glu	Gly	Ala	Pro		
Edible aquatic insects																							
Odonata	*Epophthalmia elegans*	L	65.23	9.96	2.78	6.43	5.62	7.06	3.84	9.26	0.58	3.74	1.63	50.90	11.79	6.91	3.94	10.92	4.42	5.97	5.15	60.16	[57]
	*Anax parthenope*	L	65.76	10.04	3.20	6.96	5.72	6.96	3.87	8.82	0.63	3.20	1.13	50.55	11.91	7.04	4.09	10.84	4.32	5.93	5.33	53.99	
	*Ictinogomphus rapax*	L	62.37	10.08	3.54	7.05	4.62	7.77	4.24	12.12	0.50	3.09	1.44	54.45	9.92	6.99	3.97	8.30	4.84	6.85	4.67	55.63	
	*Sinictinogomphus clavatus*	L	63.64	9.55	3.51	7.19	5.42	7.64	4.32	10.51	0.53	2.89	1.66	53.23	9.95	7.74	4.00	9.32	4.91	5.98	4.87	52.98	
	*Pantala flavescens*	L	65.18	9.78	3.30	7.20	5.97	6.48	4.06	8.61	0.65	2.87	1.32	50.26	12.47	7.38	4.07	10.73	4.64	6.00	4.45	58.16	
	*Orthetrum pruinosum*	L	71.53	9.63	3.33	7.11	5.85	6.43	3.86	8.68	0.46	2.78	1.90	50.01	12.00	7.38	4.02	11.11	4.39	5.81	5.28	54.73	
	*Crocothemis servilia*	L	65.45	6.12	3.91	7.45	7.93	6.23	5.25	2.88	2.20	5.45	3.77	51.18	8.04	8.47	4.26	10.99	5.03	7.31	4.72	51.70	[59]
	*Gomphus cuneatus*	L	64.64	6.59	7.33	3.96	6.33	7.17	4.79	3.37	0.67	6.93	4.07	51.21	4.86	6.34	4.32	14.40	5.46	7.85	5.61	50.26	
	*Lestes praemorsus*	L	46.37	6.01	6.96	4.16	8.37	7.26	4.98	3.22	5.23	6.54	2.72	55.44	8.54	6.41	4.20	13.36	4.45	7.26	5.23	36.1	
Ephemeroptera	*Ephermeterella jianghongensis*	L	66.26	5.75	5.29	8.51	5.51	6.00	4.88	3.27	-	3.33	3.39	45.93	5.75	8.71	4.55	15.21	4.96	9.15	5.74	65.54	[60]
Coleptera	*Cybister japonicus*	L	57.34	6.33	14.18	11.96	5.14	1.06	3.95	4.53	-	4.32	2.76	54.23	6.45	8.44	4.47	8.62	8.14	7.12	2.53	47.89	[61]
	*Dytiscus dauricus*	L	57.97	6.50	12.06	11.82	5.91	1.89	4.43	3.61	-	3.75	3.12	53.10	5.72	8.88	4.92	9.11	7.76	8.43	2.07	48.74	
	*Hydrophilus acminatus*	L	56.41	6.12	11.24	12.16	7.08	1.19	4.14	3.28	-	3.30	2.74	51.25	4.87	10.01	4.76	8.98	8.04	7.25	2.74	47.86	
	*H. acminatus*	L	20.37	5.76	4.38	7.59	6.89	5.11	4.26	4.33	-	6.42	2.74	47.48	5.76	9.56	3.56	9.25	8.08	10.38	5.93	42.69	[62]
Megaloptera	*Acanthacorydalis orientalis*	L	56.56	5.63	5.61	6.96	6.25	5.76	4.88	4.39	-	4.18	2.89	46.54	6.75	10.13	4.11	17.13	5.14	5.74	4.46	53.31	[60]
	*Acanthacory dalisasiatice*	A	-	7.58	5.58	9.00	7.08	9.81	4.13	10.61	-	4.02	4.21	62.01	3.90	11.73	7.69	-	-	14.34	-	52.01	[63]
	*Neochauliodes sparsus*	L	67.69	6.35	4.75	7.41	7.43	6.10	4.55	4.21	0.70	4.27	3.82	49.59	7.23	9.09	4.25	12.73	4.80	7.41	4.91	56.02	[64]
Edible terrestrial insects																							
Hymenoptera	*Polybia occidentalis nigratella*	B	61.00	5.90	4.50	7.80	7.40	5.60	4.00	3.30	0.70	3.00	5.00	47.20	5.70	8.40	4.50	12.90	7.10	6.50	6.30	-	[65]
	*Polybia parvulina*	B	61.00	6.10	4.70	7.80	7.30	5.90	4.10	3.40	0.70	3.40	5.30	48.70	5.70	7.80	4.40	13.30	7.20	6.40	6.50	-	
	*Vespa velutina*	B	-	6.10	5.50	8.70	6.10	6.60	4.20	4.20	-	4.20	2.40	47.00	4.50	6.30	6.30	20.10	6.30	5.50	6.10	37.90	[66]
	*V. mandarinia*	B	-	6.30	5.70	8.70	6.30	7.30	4.30	4.30	-	4.30	2.70	48.90	2.20	6.50	6.50	21.20	6.30	5.40	5.70	36.80	
	*V. basalis*	B	-	5.70	5.30	8.50	6.80	7.10	4.30	4.30	-	4.30	1.40	46.60	4.30	6.40	6.40	22.10	5.70	5.00	5.70	28.10	
Coleoptera	*Allomyrina dichotoma*	L	54.18	5.58	4.35	6.40	4.97	7.73	3.84	3.59	-	4.82	8.92	50.21	5.29	5.46	5.95	17.83	5.70	4.51	5.05	48.74	[32]
	*Protaetia brevitarsis*	L	44.23	6.36	4.14	5.90	4.47	8.43	3.96	4.14	-	4.65	7.51	49.54	5.34	5.77	6.51	14.15	5.72	6.23	6.72	39.16	
	*Tenebrio molitor*	L	53.22	6.61	4.45	7.57	4.52	7.75	4.11	3.96	-	6.29	7.10	52.36	5.01	6.20	4.94	12.99	5.87	8.90	3.73	44.50	
Orthoptera	*Teleogryllus emma*	A	55.65	5.85	4.30	7.93	5.23	5.23	3.84	3.58	-	4.82	7.63	48.41	7.43	7.71	5.91	13.03	5.09	9.19	3.24	49.95	
	*Gryllus bimaculatus*	A	58.32	5.94	4.01	7.38	4.50	5.07	3.72	3.40	-	4.64	9.98	48.63	6.69	6.69	5.07	11.87	6.17	10.48	3.70	53.83	
Lepidoptera	*Antheraea pernyi*	P	71.9	6.63	7.95	3.24	4.54	2.06	4.64	8.10	4.05	2.94	1.62	45.77	4.12	6.41	4.64	12.74	4.42	6.26	12.22	-	[67]
	*Bombyx mori*	P	-	5.60	5.70	8.30	7.50	5.40	5.40	5.10	9.00	2.50	6.00	60.50	6.80	10.90	4.70	14.90	4.60	5.50	4.00	-	

Note: L = larva, P = pupa, A = adult, B = brood; DM = dry matter; “-” = not determined or not estimated; ^++^ EAA: essential amino acids; we include essential amino acids (Val, Ile, Leu, Lys, Thr, Trp, Phe, His, Met) and two conditional essential amino acids (Tyr, Cys).

**Table 3:** Fatty acid composition of edible aquatic insects and selected edible terrestrial insects.

Order	Species	Developmental Stage	Lipid %	Fatty Acid Composition (% of Total Fatty Acids)										Reference
				C14:0	C16:0	C18:0	SFA	C18:1	MUFA	C18:2	C20:4	C20:5	PUFA	
Aquatic insects														
Odonata	*Epophthalmia elegans*	L	9.14	1.67	21.88	7.85	33.73	16.97	32.84	4.46	5.02	6.11	21.05	[47]
	*Anax parthenope julius*	L	11.06	1.18	24.41	7.00	34.68	17.65	27.85	7.01	3.73	3.08	27.85	
	*Ictinogomphus rapax*	L	10.59	2.07	19.30	6.98	30.11	19.01	35.75	7.49	1.89	5.08	20.33	
	*Pantala flavescens*	L	10.4	0.75	21.6	8.55	32.3	11.98	33.07	9.59	1.91	9.44	27.51	
	*Inictinogomphus clavatus*	L	11.9	0.89	24.61	7.74	34.43	20.58	42.28	6.66	1.24	3.87	17.75	
	*Orthetrum pruinosum neglectum*	L	5.72	2.34	17.57	7.65	34.97	6.85	31.53	5.77	6.70	7.83	26.70	
Diptera	*Stictochironomus pictulus*	L	-	4.70	16.10	6.20	34.00	11.00	49.50	6.70	1.00	3.60	14.30	[68]
	*Anopheles albimanus*	L	-	1.94	28.03	7.76	44.50	22.26	31.13	7.59	3.07	4.19	24.40	[69]
	*A. vestitipennis*	L	-	1.86	21.82	6.62	37.39	22.06	36.16	12.28	2.85	2.47	26.43	
	*A. darlingi*	L	-	1.26	25.42	6.64	39.53	22.44	30.76	18.66	2.46	2.39	29.69	
Coleoptera	*Cybister japonicus*	A	27.66	3.41	12.01	5.18	27.56	35.61	49.94	9.82	3.55	3.96	22.52	[61]
	*Dytiscus danmcus*	A	27.56	2.86	21.63	2.45	35.1	29.94	46.95	6.53	3.54	4.08	19.46	
	*Hydrophilus aoninatus*	A	31.86	9.16	19.09	-	65.2	1.83	3.81	1.98	-	-	30.92	
Terrestrial insects														
Orthoptera	*Gryllus bimaculatus*	L	28.90	-	25.44	8.74	34.67	25.86	26.54	37.05	-	-	38.79	[70]
	*Ruspolia differens*	A	48.20	0.90	31.50	5.50	38.30	24.60	26.60	31.20	-	-	34.40	[53]
Coleoptera	*Tenebrio molitor*	L	31.97	4.45	21.33	7.92	33.70	35.83	37.80	22.83	0	0	22.94	[71]
Hymenoptera	*Apis mellifera*	L	4.90	2.40	37.30	11.80	51.80	47.50	48.20	0	-	-	0	[72]
	*Vespa mandarinia*	B	20.20	2.50	21.30	5.00	30.70	27.70	29.20	33.70	-	-	40.10	
Lepidoptera	*Bombyx mori*	P	32.20	0.10	24.20	4.50	24.30	26.00	27.70	7.30	-	-	36.30	[73]

Note: L = larva, P = pupa, A = adult; “-” = not determined or not estimated; oil. SFA = saturated fatty acids, MUFA = monounsaturated fatty acids, PUFA = polyunsaturated fatty acids.

**Table 4:** Mineral content [mg/kg, DM] of selected edible aquatic insects and edible terrestrial insects.

Species	Developmental Stage	Ca	Mg	K	Na	Fe	P	Mn	Cu	Zn	Se	References
Edible aquatic insects												
*Anax parthenope*	L	124.960	116.900	1591.900	1339.760	158.210	-	6.790	4.180	74.770	0.193	[57,58]
*Epophthalmia elegans*	L	90.110	101.800	1350.790	1372.490	22.640	-	12.050	2.460	40.410	0.223	[57,58]
*Crocothemes servillia*	L	865.000	370.000	2680.000	14,100.000	113.000	-	-	19.000	93.000	-	[30]
*Lethocerus indicus*	L & A	960.000	703.300	1700.000	8550.000	4100.000	-	-	11.000	295.000	-	[30]
*Laccotrephes maculatus*	L & A	665.000	460.000	5500.000	15,000.000	250.000	-	-	137.000	231.500	-	[30]
*Cybister tripunctatus*	A	277.000	336.000	6430.000	3050.000	73.000	-	-	51.000	57.500	-	[30]
*C. japonicus*	A	3602.820	774.520	6722.650	2251.760	148.160	5809.000	8.700	29.370	93.990	0.360	[58,74]
*Hydrophilus olivaceous*	A	243.000	990.000	3900.000	8160.000	4610.000	-	-	17.000	118.000	-	[30]
*Hydrous acuminatus*	A	106.700	109.200	1807.700	-	83.200	1905.100	-	-	29.000	-	[62]
Edible terrestrial insects												
*Gryllus bimaculatus*	A	1660.850	1073.750	8607.500	3649.450	81.800	11,696.000	66.300	36.250	232.650	0.490	[32,70]
*Acheta domesticus*	L & A	1261.150	1040.550	13,318.700	5122.900	77.650	10,291.150	38.100	21.200	257.400	0.500	[75]
*Teleogryllus emma*	A	1935.400	1524.800	8955.000	2782.300	107.500	10,854.000	58.600	21.900	184.700	-	[32]
*Tenebrio molitor*	L	504.800	2450.800	8212.375	1047.125	98.393	8282.233	14.170	18.138	116.660	0.377	[32,75,76]
*Zophobas morio*	L	420.400	1182.900	7505.900	1128.300	39.200	5629.500	10.200	8.600	72.900	0.300	[75]
*Protaetia brevitarsis*	L	2585.600	3276.000	20,014.000	2116.000	162.000	11,404.000	58.900	18.200	118.900	-	[32]
*Allomyrina dichotoma*	L	1234.000	2835.600	12,491.000	1483.800	142.600	8606.900	86.400	14.300	102.600	0.064	[32,58]
*Anoplophora chinensis*	L	269.300	1881.000	5647.000	92.850	131.280		35.020	8.980	223.640	0.050	[76]
*Galleria mellonella*	L	585.500	761.400	5325.300	397.600	50.400	4698.800	3.100	9.200	61.200	0.300	[75]
*Bombyx mori*	L & P	1023.100	2878.600	18,265.900	2745.700	95.400	13,699.400	24.900	20.800	177.500	0.575	[58,75]
*Antheraea pernyi*	P	234.000	707.000	4020.000	57.400	13.400	-	2.200	2.900	30.600	0.210	[77]
*Vespa velutina*	L & P	388.000	639.000	7516.000	104.000	100.000	5612.000	6.000	22.000	72.000	-	[66]
*Polyrhachis vicina*	A	785.500	664.500	-	-	858.500	4028.500	291.000	21.500	147.500	0.335	[78]

Note: L = larva, P = pupa, A = adult; “-” = not determined or not estimated; Mineral compositions [mg/kg] of calcium (Ca), magnesium (Mg), potassium (K), sodium (Na), iron (Fe), phosphorus (P), manganese (Mn), copper (Cu), zinc (Zn), and selenium (Se) were analyzed based on dry matter (DM); if different values were involved in multiple literatures, the mean value was listed.

Environmental factors seem to be the main factor determining the mineral element contents of aquatic insects. It is reported that, despite their body size, dragonflies had good ability to absorb environmental metallic elements (Hg, As, Pb, Cr, Cu, Cd, Ni, Se, Al, and Au) [79], thus they are good ambient heavy metal content indicators.

### 3.4. Chitin and Chitosan

Chitin is a significant biopolymer [80], and chitosan is formed by the deacetylation of chitin. They could be used in food, biomedical and cosmetic industries, and for wastewater treatment and textiles. Up to date, crustacean shells from the marine food industry provide us with the chief commercial sources of chitin and chitosan [80,81]. Due to the COVID-19 pandemic, biopolymer materials have been increasingly in demand, and the market for chitin and chitosan is growing steadily [81].

Chitin is found throughout the exoskeletons of most insects. In recent years, some terrestrial insect species have been investigated as alternative chitin sources [82,83,84], since insects were considered as a potential resources of food. As compared to the existing sources, the extraction of chitin and chitosan from insects is simple, requires less chemical consumption and time, and they can be extracted in a higher yield [81]. Moreover, insect-derived chitin and chitosan have numerous biological effects, such as antioxidant and antibacterial activities with substantial rheological properties [82,83]. In Table 5, the percentages of chitin and chitosan from terrestrial insects are species specific and are in the ranges of 2.59–36.80% and 16.00–96.35%, respectively. However, for aquatic insects, far fewer literature studies are reported. The contents of chitin in aquatic insects have almost no differences with terrestrial insects; the former may also be used as alternative chitin sources [85]. More extensive and comparative research of chitin and chitosan between aquatic insects and terrestrial insects is needed.

### 3.5. Active Substances and Healthcare

Edible insects are considered to have superior health benefits, due to their high quantities of nutrients, such as essential amino acids, omega-3 and omega-6 fatty acids, vitamin B12, iron, and zinc [2,72,96]. In addition to the health benefits of edible insect nutrients, the rich active substances in edible aquatic insects have also attracted attention [97]. Edible aquatic insects, such as dragonflies, water strider, and whirligig beetle, have been used in healthcare or for treating human diseases since the ancient times [98,99,100], especially in the countries of East Asia [101], e.g., China, Japan, and South Korea. The theory of traditional Chinese medicine is that the larva of aquatic insects has the effects of boosting the kidney, nourishing essence, moisturizing the lungs, and relieving coughs [102]. They can be used alone or in combination with other materials for medical applications; for example, the whole body of a dried adult dragonfly can be used to treat impotence and nocturnal emission, sore throat, and whooping cough [103]. It is believed that *Cybister tripunctatus,* or *C. japonicus,* can reinforce kidney function and invigorate the circulation of blood in human beings [102]. These traditional practices of edible aquatic insects also provide ideas for the exploitation of modern drugs [104]; for example, the methanol extract of *C. tripunctatus* displays strong antioxidant activity at a concentration of 110 μg/mL [30]. It is reported that the water and liposoluble extracts of the giant water bugs *L. indicus* have negligible values of antioxidant capacity compared to the extracts of terrestrial insects, such as grasshoppers, silkworm, and crickets, in vitro [105].

The bioactive ingredients in insects have different sources depending on the species [106,107,108]. Some natural toxins are produced by insects in their special organs, such as bee venom and cantharidin [109,110]. The giant water bug *L. indicus*, which is consumed as both a medicinal and edible insect [111], could produce complex chemicals in its odorous gland [112,113]. On the other hand, some other components from insects are enriched and accumulated from the plants, fungi, and algae that they feed on. Phytophagous insects have evolved unique systems to keep the secondary metabolites taken up from the plant in their bodies [114] and utilize them for escaping from predators [115,116], and these accumulated secondary metabolites have a certain value in drug development [117,118]. However, exploration of the source of functional substances of aquatic insects is still very limited. This is an aspect worth exploring further.

## 4. Safety in Utilization of Edible Aquatic Insects

### 4.1. Contaminant

Aquatic insects, as environmental indicators, have attracted extensive attention for a long time, and their related research is very rich. A water body is an open environment, which readily gathers various substances through water flow, soil, etc. Due to the close relationship between aquatic organisms and water, aquatic insects easily accumulate various pollutants, including heavy metals, pathogens, pesticides, and so on. These contaminants may enter the human body through the food chain, which will cause harm to human health. We should draw more attention to them. This part mainly indicates the possible risks of the utilization of aquatic insects, but the relevant processes and pollution mechanisms are beyond the scope of this review.

Heavy metals. Heavy metal pollution in the freshwater ecosystem poses a great threat to the growth of aquatic insects [119,120]; therefore, there is a growing concern that it will eventually affect the health of humans [121,122]. Up to date, over 33 metallic elements of aquatic edible insects have been detected, and heavy metals, such as Hg, Pb, Cd, and Cr, have attracted serious concern [123]. Most of the metals in nature will enter the water, and the aquatic ecosystem plays an important role in the transfer and circulation of metals. Insects could absorb metal elements and accumulate a higher concentration of them than the environment [124]. The degree of metal element absorption by aquatic insects is related to environmental factors; for example, aquatic insects collected near wastewater treatment plants, or near mines, may show higher metallic element concentrations when compared with other sites [125,126]. Meanwhile, the mercury content in carnivorous insects was generally higher than that in herbivorous insects, and the mercury content in aquatic insects was much higher than that in terrestrial insects (*t*-test, *p* < 0.01), with the investigation of 42 insect species from Yunnan, China [58]. Many studies [121,127,128,129,130,131,132] have indicated that the larvae of aquatic insects accumulate metals in aquatic ecological environments and retain them until the adult stage. After being preyed on by bats, spiders, birds, fish, and so on, they become the connector of the aquatic and terrestrial food chains, and, thus, bring metal elements into the terrestrial food chain. This suggests that there are the same risks in the process of human consumption of aquatic insects.

Pathogen. Aquatic insects are important vectors (e.g., malaria) of environmental pathogens. Mainly originating from the Japanese encephalitis (JE) virus, the viral encephalitis in Southeast Asia was detected in *Culex gelidus* in 1976 [133]. Aquatic insects are possible vectors of *Mycobacterium ulcerans*, which causes chronic skin ulcers in tropical countries [134]. Two conditioned pathogens, *Lelliottia amnigena* and *Citrobacter freundii,* were detected from edible aquatic insects of the genus *Cybister* [135].

Pesticide. Pesticide residues in food can cause damage to the human nervous and reproductive systems, interfere with the normal operation of the human immune or endocrine system, and even induce cancer. Pesticides could enter aquatic insects through contact or feeding [136]. The impact of pesticides reaching streams and potentially harming the aquatic juvenile stages has drawn extensive concerns when investigating the effects of insecticides on freshwater insects [137,138]. There are few studies on the effects of pesticide residues in insects on human health.

### 4.2. Purine Derivatives and Uric Acid

Uric acid serves as an antioxidant, and is important for protecting human blood vessels. It is metabolized from purines [139], which are important nucleic acid components in all organisms. However, increasing numbers of people are suffering from hyperuricemia, gout, and other disease caused by frequent and high intake of purine-rich and protein-rich foods, which enhances serum uric acid levels. An important way to treat people with hyperuricemia or gout is dietary restriction of purine-rich foods [140,141].

The concentration of purine derivative and uric acid in edible insects varies, primarily with species and gender (in some cases) [140,142,143]. Though no common characteristics of purine and uric acid contents in different edible insects or at different life cycle stages of the same insect were indicated, much lower uric acid levels are detected in aquatic insects than in terrestrial insects [143]. A possible reason for this could be the extremely moist environments that aquatic insects live in, where they can excrete large quantities of ammonia. However, for most terrestrial insects, loss by excretion has to be minimized because water conservation is essential throughout their life cycles. Hence, more than 80% of the total nitrogenous materials excreted from most terrestrial insects are uric acid [144].

### 4.3. Allergy

Seafood is an important origin of allergies, and, thus, has attracted attention as a food safety issue. Similarly, most known edible insect allergens have cross-reactivity with homologous proteins in shellfish [145]; therefore, enough attention should be paid to the insect allergy too. At present, all the reported cases of insect allergies are from terrestrial insects, such as silkworm, mealworm, caterpillars, wasps, grasshoppers, cicada, and bees [146].

## 5. Discussion

### 5.1. Characteristics of Nutrition in Aquatic Insects

There is much less available information on aquatic insects, especially on their nutritional value, when compared to terrestrial insects. In this paper, we summarize some results of the research on aquatic insect nutrition. It should be noted that, for the nutritional composition, there must be variations, even in the same species, attributed to some internal (i.e., feed, developmental and physiological state of the samples analyzed) and external (i.e., season, geographic location, techniques employed, etc.) factors [30,147,148,149].

Both aquatic insects and terrestrial insects have high protein contents, with some differences in amino acid compositions, kinds of restricted amino acids, and the dominant amino acids. It should be encouraged that proteins from different sources (e.g., aquatic insects and terrestrial insects) are used simultaneously, so as to complement each other and improve the biological value of insect proteins.

A remarkable feature of aquatic insects is the rich, highly unsaturated fatty acid (HUFA) content, which is much higher than in terrestrial insects, especially the EPA content. This big difference might be explained by their food webs. The food of terrestrial insects, such as vascular plants, usually only contains the HUFA precursor α-Linolenic acid (ALA) [150], while aquatic insects feed on freshwater algae and diatoms, which are particularly rich in HUFA and EPA [151].

The living environment is a main factor affecting the selenium content in insects. The reason for the significant effect of aquatic habitat on the selenium content of insects may be related to the characteristics of selenium. Selenium in soil can be leached into the aquatic ecosystem; therefore, aquatic organisms are more easily exposed to selenium than terrestrial organisms [152]. Aquatic organisms usually have a larger selenoprotein group, while terrestrial organisms have a significantly smaller selenoprotein group. It is speculated that terrestrial organisms gradually lost their selenoprotein-coding genes, or replaced the Sec with cysteine in the original selenoprotein-coding genes [153,154]. This may help explain why the selenium content of aquatic insects is higher than that of terrestrial insects [58].

### 5.2. Edible Aquatic Insects Resources and Farming

In order to protect wild resources and control the quantity and quality of products, it is very necessary to cultivate insects when we promote insect consumption. Edible insect farming has already taken off all around the world. The farming technology of some terrestrial insect food or feed species, such as mealworm, black soldier flies, crickets, and houseflies, has largely advanced, and their yields are very high and sustainable nowadays [21]. However, there is little information about rearing aquatic insects, especially edible aquatic insects.

Aquatic insects should be cultivated on a large scale for the following reasons. Firstly, excessive collection of natural resources will lead to a reduction in aquatic insect resources and regional extinction of species. The majority of edible insects commercially consumed are mainly collected from nature [23]. Due to the lack of rearing technology and boom of traditional customs, collecting edible aquatic insects from the wild is much more prevalent than collecting terrestrial insects. However, the overexploitation of several species in Mexico has led to a subsequent decrease in the population [155], and the uncontrolled harvesting of wild populations may prove unsustainable [22]. Fortunately, the production patterns of edible insects in Thailand and Lao People’s Democratic Republic have changed to semi-domestication and insect farming, besides the traditional harvesting of insects from wild habitats [156]. Secondly, the artificial cultivation of insects could avoid the disturbance from pollutants. As mentioned previously, a water body is an open environment, which is easily disturbed by pollutants, affecting the population growth and consumption safety of aquatic edible insects. Thirdly, artificial cultivation can improve the nutritional quality of aquatic insects through feeding technology [157], such as to attain a beneficial n-6/n-3 ratio, or to better meet the nutritional demands of the consumer. Finally, population outbreaks do not theoretically easily happen in aquatic insects [158]; therefore, it is necessary to expand the population by artificial promotion.

In addition, the development of aquatic edible insect cultivation technology can be supported by several aspects of experience. Historically, there have been some practices of raising insects all over the world. Semi-domestication also has the potential to promote the yield of insects greatly [22]. A famous example of semi-domestication from Mexico showed that eggs of semi-cultivated aquatic Hemiptera were used by local people, through water management and to provide egg laying sites [1]. In modern times, the breeding of aquatic insects is not a blank, but has accumulated technologies and experience in species from several orders [159,160,161]. Moreover, the successful breeding of terrestrial insects (e.g., crickets, mealworms, and black solider flies) can provide a reference for the breeding of aquatic insects [19]. In recent years, some species from Hemiptera, Coleoptera, and Megaloptera have had some success in farming, e.g., *L. indicus* in Thailand, and predacious diving beetle [162,163] and hellgrammites [25,26] in China.

Nevertheless, the aquaculture of aquatic insects still needs to face the problems of high input costs, such as water quality, energy, and feed [18,164]. It is suggested that different types of aquatic insect breeding technologies should be developed and utilized, according to market demand, product value, regional conditions, and other factors, and regional planning should be performed for aquatic insect breeding. For example, for the species with high market recognition and health care function, priority should be given to the development of automatic three-dimensional breeding, and most aquatic edible insects should be cultivated with semi-artificial technology, or artificial promotion and management technology. Similarly, a large area and protected water source can harvest a large number of aquatic insect products. On the contrary, such appropriate conditions can guide more people to breed in this area, so as to improve the technical maturity. Of course, the cultivation of aquatic insects will also help to prevent environmental pollution; for example, in order to harvest aquatic insects raised in the farmland system, excessive pesticides and fertilizers cannot be applied, which reduces the pressure of environmental pollution.

### 5.3. Enrich the Use of Aquatic Insects

Edible aquatic insects also have the potential to serve human health and feed domestic animals. The utilization of aquatic insects is small in scale, but the prospects are obvious, and the development of new uses can increase its attraction. The active substances of aquatic insects are specific products in aquatic environments, which are worthy of further study.

It is a valuable and innovative proposal to use edible aquatic insects as feed, and some species, such as mosquito larvae, could provide a convenient source of feed [19,165]. Mosquitos have the distinct biological characteristics of a short lifecycle, high occurrence densities, and wide distribution. In one case, the larvae of chironomid from chicken manure were used for fish food, both in local areas and North America [159]. Aquatic insects with high HUFA are being designed to be aquafeeds and farmed marine fishes, for meeting the demand of the HUFA of fishes [19,166].

## 6. Conclusions

Edible insects are promising safe food that have high potential to be more sustainable and more equitable at the global scale. Resources of insects, both from terrestrial and aquatic environments, should be encouraged. Although the uses of aquatic insects as food, as feed, and in healthcare were ignored to a certain extent, edible aquatic insects have great potential for utilization.

There has been the discovery of a few characteristics of edible aquatic insects with limited documents, compared with terrestrial insects. Two sources of edible insects have a high content of protein, calcium, iron, and zinc, and similar contents of chitin. There is no doubt that they are all excellent biological resources. The living environment seems to be the main factor for the origin of the difference between aquatic and terrestrial insects. A water body is an open environment; therefore, aquatic organisms are more easily exposed to various substances. Compared with edible terrestrial insects, aquatic insects usually have different limiting amino acids and a higher content of delicious amino acids, and contain highly unsaturated omega-3 fatty acids and some long-chain polyunsaturated fatty acids, lower uric acid, much higher amounts of calcium and selenium, and a larger selenoprotein group, and face higher risk of exposure to contaminants (e.g., heavy metal, pesticides). More studies investigating the distinct differences between aquatic and terrestrial insects are needed for better conservation and utilization of these insects.

## Figures and Tables

**Table 1:** The species information of edible aquatic insects.

Order	Number	Feeding Habits	Edible Stage	Mainly Edible Family—Genus (Edible Species Number)	Edible Country (Edible Species Number)
Ephemeroptera	11	phytophagous	naiad, adult	Ephemeridae—Ephemera (3), Baetidae—Cloeon (2)	India (3), Mexico (2), Malawi (2), Japan (2), China, Papua New Guinea, Kenya, Malawi, Tanzania, Uganda
Odonata	72	predatism	naiad, adult	Libellulidae—Sympetrum (8), Libellulidae—Orthetrum (7), Aeschnidae—Rhionaeschna (4), Libellulidae—Neurothemis (3), Libellulidae—Trithemis (3), Aeschnidae—Anax (3),	India (14), China (13), Thailand (13), Indonesia (12), Venezuela (10), Japan (10), Ecuador (4), Mexico (3), Laos (3), Madagascar (2), Myanmar (2), Vietnam (2), USA, Italy, South Korea, D.R.Congo
Plecoptera	11	omnivorous	naiad	Pteronarcyidae—Pteronarcys (4)	Japan (6), USA (4), India (2)
Hemiptera	68	predatism	egg, naiad, adult	Nepidae—Laccotrephes (7), Belostomatidae—Lethocerus (6), Nepidae—Ranatra (5) Belostomatidae—Abedus (3), Belostomatidae—Diplonychus (3), Belostomatidae—Sphaerodema (3), Corixidae—Corisella (3), Corixidae—Graptocorixa (3)	Mexico (17), Thailand (17), China (8), Japan (8), India (7), Venezuela (4), Madagascar (4), Laos (4), USA (3), Myanmar (3), Vietnam (2), D.R. Congo, Cameroon, Zambezian region, Malaysia, Mali, Singapore, Sri Lanka, Republic of Congo, Togo, Malawi, South Korea
Megaloptera	15	predatism	larva	Corydalidae—Acanthacorydalis (6), Corydalidae—Corydalus (4)	China (9), Japan (2), Peru (2), Mexico, Colombia, Venezuela
Coleoptera	108	predatism	larva, pupa, adult	Dytiscidae—Cybister (32), Hydrophilidae—Hydrophilus (14), Dytiscidae—Dytiscus (6), Dytiscidae—Rhantus (6), Dytiscidae—Laccophilus (5), Hydrophilidae—Tropisternus (5), Gyrinidae—Gyrinus (4)	Mexico (35), China (26), Japan (22), Thailand (21), India (11), Madagascar (10), Laos (9), Vietnam (7), Myanmar (6), Senegal (4), North Korea (3), Sri Lanka(3), Benin (3), Malaysia (3), Togo (3), Chile (3), Sabah (2), Cambodia (2), South Korea (2), USA (2), Indonesia (2), Cameroon (2), Turkey (2), Peru (2), Australia (2), Sierra Leone, D.R.Congo, Gabon, Panama
Diptera	27	saprophagous	larva, pupa	Chaoboridae—Chaoborus (4), Tipulidae—Tipula (4), Ephydridae—Ephydra (3), Simuliidae—Simulium (3)	USA (7), Uganda (6), Mexico (5), Venezuela (2), Japan (2), Kenya (2), Tanzania (2), Brazil, China, Colombia, Malawi, N. Am., Nearctic, Sri Lanka, Thailand
Trichoptera	17	phytophagous	larva	Stenopsychidae—Stenopsyche (3)	Japan (12), Venezuela (4), Mexico (2), Colombia

**Table 5:** Chitin and chitosan from aquatic insect and selected edible terrestrial insects.

Order	Species	Developmental Stage	Yield of Chitin (%)	Yield of Chitosan (%)	Reference
Aquatic insects					
Coleoptera	*Agabus bipustulatus*	-	14.00–15.00	71.00	[85]
	*Hydrophilus piceus*	-	19.00–20.00	74.00	
Odonata	*Anax imperator*	L	11.00–12.00	67.00	
Hemiptera	*Notonecta glauca*	-	10.00–11.00	69.00	
	*Ranatra linearis*	-	15.00–16.00	70.00	
Terrestrial insects					
Lepidoptera	*Bombyx mori*	P	2.59–4.23	73.00–96.35	[86]
Coleoptera	*Catharsius molossus*	A	24.00	70.83	[87]
Orthoptera	*Pterophylla beltrani*	-	11.80	58.80	[88]
	*Brachytrupes portentosus*	-	4.30–7.10	55.81–81.69	[89]
	*Calliptamus barbaru*	A	20.50	74.00–75.00	[90]
	*Oedaleus decorus*	A	16.50	75.00–76.00	
Hymenoptera	*Apsis mellifera*	A	19.00–36.80	16.00–30.00	[91]
Diptera	*Musca domestica*	P	8.02	73.19	[92]
	*Hermetia illucens*	L	7.00	32.00	[93]
	*Drosophila melanogaster*	A	7.85	70.91	[94]
Blattodea	*Periplaneta americana*	-	12.17	59.82	[95]

Note: L = larva, P = pupa, A = adult, “-” = not sure; the yield of chitosan is calculated from chitin, or degree of deacetylation.

## Data Availability

Not applicable.

## Supplementary Materials

The following are available online at https://www.mdpi.com/article/10.3390/foods10123033/s1, Table S1: full list of named edible aquatic insects.

## References

[1] Van Itterbeeck J., van Huis A. (2012). Environmental manipulation for edible insect procurement: A historical perspective. J. Ethnobiol. Ethnomed..

[2] Feng Y., Chen X.-M., Zhao M., He Z., Sun L., Wang C.-Y., Ding W.-F. (2017). Edible insects in China: Utilization and prospects. Insect Sci..

[3] Kim T.-K., Yong H.I., Kim Y.-B., Kim H.-W., Choi Y.-S. (2019). Edible Insects as a Protein Source: A Review of Public Perception, Processing Technology, and Research Trends. Food Sci. Anim. Resour..

[4] Raheem D., Carrascosa C., Oluwole O.B., Nieuwland M., Saraiva A., Millán R., Raposo A. (2018). Traditional consumption of and rearing edible insects in Africa, Asia and Europe. Crit. Rev. Food Sci. Nutr..

[5] Ramos-Elorduy J. (2009). Anthropo-entomophagy: Cultures, evolution and sustainability. Entomol. Res..

[6] Meyer-Rochow V.B. (1973). Edible insects in three different ethnic groups of Papua and New Guinea. Am. J. Clin. Nutr..

[7] Séré A., Bougma A., Ouilly J.T., Traoré M., Sangaré H., Lykke A.M., Ouédraogo A., Gnankiné O., Bassolé I.H.N. (2018). Traditional knowledge regarding edible insects in Burkina Faso. J. Ethnobiol. Ethnomed..

[8] Hlongwane Z.T., Slotow R., Munyai T.C. (2020). Indigenous Knowledge about Consumption of Edible Insects in South Africa. Insects.

[9] Mwangi M.N., Oonincx D.G.A.B., Stouten T., Veenenbos M., Melse-Boonstra A., Dicke M., Van Loon J.J.A. (2018). Insects as sources of iron and zinc in human nutrition. Nutr. Res. Rev..

[10] Oonincx D.G.A.B., van Itterbeeck J., Heetkamp M.J.W., Brand H.V.D., van Loon J.J.A., van Huis A. (2010). An Exploration on Greenhouse Gas and Ammonia Production by Insect Species Suitable for Animal or Human Consumption. PLoS ONE.

[11] Gahukar R.T., Dossey A.T., Morales-Ramos J.A., Rojas M.G. (2016). Edible Insects Farming: Efficiency and Impact on Family Livelihood, Food Security, and Environment Compared With Livestock and Crops. Insects Sust Food Ingredients.

[12] Hlongwane Z.T., Slotow R., Munyai T.C. (2020). Nutritional Composition of Edible Insects Consumed in Africa: A Systematic Review. Nutrients.

[13] Patel S., Suleria H.A.R., Rauf A. (2019). Edible insects as innovative foods: Nutritional and functional assessments. Trends Food Sci. Technol..

[14] Huis A.v., Itterbeeck J.V., Klunder H., Mertens E., Halloran A., Muir G., Vantomme P. (2013). Edible Insects: Future Prospects for Food and Feed Security.

[15] Koroiva R., Pepinelli M., Del-Claro K., Guillermo R. (2019). Distribution and Habitats of Aquatic Insects. Aquatic Insects: Behavior and Ecology.

[16] Williams D.D., Feltmate B.W. (2017). Aquatic Insects.

[17] Kitsa K. (1989). Contribution des insectes comestibles a l’amélioration de la ration alimentaire au Kasai Occidental. Zaire-Afrique.

[18] Yen A. (2015). Insects as food and feed in the Asia Pacific region: Current perspectives and future directions. J. Insects Food Feed.

[19] Williams D.D., Williams S.S. (2017). Aquatic Insects and their Potential to Contribute to the Diet of the Globally Expanding Human Population. Insects.

[20] Hotaling S., Kelley J., Frandsen P. (2020). Aquatic Insects Are Dramatically Underrepresented in Genomic Research. Insects.

[21] Williams D., Williams S., van Huis A. (2021). Can we farm aquatic insects for human food or livestock feed?. J. Insects Food Feed.

[22] Macadam C., Stockan J. (2017). The diversity of aquatic insects used as human food. J. Insects Food Feed.

[23] Feng Y., Zhao M., Ding W., Chen X. (2020). Overview of edible insect resources and common species utilisation in China. J. Insects Food Feed.

[24] Tanaka R., Oda M. (2009). Cyclic Dipeptide of D-ornithine obtained from the dobsonfly, *Protohermes grandis* Thunberg. Biosci. Biotechnol. Biochem..

[25] Cao C. (2016). Rearing hellgrammites for food and medicine in China. J. Insects Food Feed.

[26] Shi Z.X. (2008). A research on artificial culture of climbing-sand worms-panxi special aquatic organisms based on computer control. J. Xichang Coll. (Nat. Sci. Ed.).

[27] Jongema Y. List of Edible Insect Species of the World. https://www.wur.nl/en/Research-Results/Chair-groups/Plant-Sciences/Laboratory-of-Entomology/Edible-insects/Worldwide-species-list.htm.

[28] Mitsuhashi J. (2016). Edible Insects of the World.

[29] Wang C.Y., Zhao M., Wang J.D., Jiang Y.Y., He Z., Feng Y. (2018). Molecular identification of a new species of edible dragonfly. Biot. Resour..

[30] Shantibala T., Lokeshwari R.K., Debaraj H. (2014). Nutritional and antinutritional composition of the five species of aquatic edible insects consumed in Manipur, India. J. Insect Sci..

[31] Nurhasan M., Maehre H.K., Malde M.K., Stormo S.K., Halwart M., James D., Elvevoll E.O. (2010). Nutritional composition of aquatic species in Laotian rice field ecosystems. J. Food Compos. Anal..

[32] Ghosh S., Lee S.-M., Jung C., Meyer-Rochow V.B. (2017). Nutritional composition of five commercial edible insects in South Korea. J. Asia-Pac. Entomol..

[33] Bergeron D., Bushway R.J., Roberts F.L., Kornfield I., Okedi J., Bushway A.A. (1988). The nutrient composition of an insect flour sample from Lake Victoria, Uganda. J. Food Compos. Anal..

[34] Okedi J. (1992). Chemical evaluation of Lake Victoria lakefly as nutrient source in animal feeds. Int. J. Trop. Insect Sci..

[35] FAO, WHO, UNU (2007). Protein and Amino Acid Requirements in Human Nutrition: Report of a Joint WHO/FAO/UNU Expert Consultation.

[36] Bukkens S. (1997). The nutritional value of edible insects. Ecol. Food Nutr..

[37] Zielińska E., Baraniak B., Karaś M., Rybczyńska-Tkaczyk K., Jakubczyk A. (2015). Selected species of edible insects as a source of nutrient composition. Food Res. Int..

[38] Ekpo K.E. (2011). Effect of processing on the protein quality of four popular insects consumed in Southern Nigeria. Arch. Appl. Sci. Res..

[39] De Guevara O.L., Padilla P., García L., Pino J.M., Ramos-Elorduy J. (1995). Amino acid determination in some edible Mexican insects. Amino Acids.

[40] Aguilar J.G.d.S. (2021). An overview of lipids from insects. Biocatal. Agric. Biotechnol..

[41] Hixson S.M., Sharma B., Kainz M.J., Wacker A., Arts M. (2015). Production, distribution, and abundance of long-chain omega-3 polyunsaturated fatty acids: A fundamental dichotomy between freshwater and terrestrial ecosystems. Environ. Rev..

[42] Bell J., Ghioni C., Sargent J.R. (1994). Fatty acid compositions of 10 freshwater invertebrates which are natural food organisms of Atlantic salmon parr (*Salmo salar*): A comparison with commercial diets. Aquaculture.

[43] Béligon V., Christophe G., Fontanille P., Larroche C. (2016). Microbial lipids as potential source to food supplements. Curr. Opin. Food Sci..

[44] Twining C.W., Brenna J.T., Lawrence P., Winkler D.W., Flecker A.S., Hairston N.G. (2019). Aquatic and terrestrial resources are not nutritionally reciprocal for consumers. Funct. Ecol..

[45] Popova O.N., Haritonov A.Y., Sushchik N.N., Makhutova O.N., Kalachova G.S., Kolmakova A.A., Gladyshev M.I. (2017). Export of aquatic productivity, including highly unsaturated fatty acids, to terrestrial ecosystems via Odonata. Sci. Total Environ..

[46] Twining C.W., Shipley J.R., Winkler D.W. (2018). Aquatic insects rich in omega-3 fatty acids drive breeding success in a widespread bird. Ecol. Lett..

[47] Jiang Y.Y., He Z., Zhao M., Wang C.Y., Sun L., Feng Y. (2017). Oil content and fatty acid composition of six kinds of common edible dragonfly naiads. China Oils Fats.

[48] Hanson B. (1983). Lipid Content and Fatty Acid Composition of Aquatic Insects: Dietary Influence and Aquatic Adaptation. Ph.D. Thesis.

[49] Cerritos R. (2009). Insects as food: An ecological, social and economical approach. CAB Rev. Perspect. Agric. Vet. Sci. Nutr. Nat. Resour..

[50] Banjo A.D., Lawal O.A., Songonuga E. (2006). The nutritional value of fourteen species of edible insects in southwestern Nigeria. Afr. J. Biotechnol..

[51] Kinyuru J.N., Mogendi J.B., Riwa C.A., Ndung’u N.W. (2015). Edible insects-A novel source of essential nutrients for human diet: Learning from traditional knowledge. Anim. Front..

[52] Elser J.J., Fagan W.F., Denno R.F., Dobberfuhl D.R., Folarin A., Huberty A.F., Interlandi S.J., Kilham S.S., McCauley E., Schulz K. (2000). Nutritional constraints in terrestrial and freshwater food webs. Nature.

[53] Kinyuru J.N., Kenji G.M., Muhoho S.N., Ayieko M. (2009). Nutritional potential of longhorn grasshopper (*Ruspolia differens*) consumed in Siaya District, Kenya. J. Agric. Sci. Technol..

[54] Adeduntan S.A. (2005). Nutritonal and Antinutritional Characteristics of Some Insects Foragaing in Akure Forest Reserve Ondo State, Nigeria. J. Food Technol..

[55] Dunkei F.V. (1996). Nutritional value of various insects per 100 grams. Food Insect Newsl..

[56] Omotoso O. (2006). Nutritional quality, functional properties and anti-nutrient compositions of the larva of *Cirina forda* (Westwood) (Lepidoptera: Saturniidae). J. Zhejiang Univ. Sci. B.

[57] Jiang Y.Y., Zhao M., He Z., Wang C.Y., Sun L., Feng Y. (2017). Nutrition composition and evaluation of six edible dragonfly naiads. Biot. Resour..

[58] Wang J.D., Wang C.Y., Zhao M., He Z., Sun L., Feng Y. (2019). Contents of Mercury and Selenium in Common Edible and Medicinal Insects in Yunnan and Their Correlation Analysis. J. Yunnan Agric. Univ. Nat. Sci..

[59] Feng Y., Chen X.M., Wang S.Y., Ye S.D., Chen Y. (2001). Three Edible Odonata Species and Their Nutritive Value. For. Res..

[60] Feng Y., Chen X.M., Zhao M. (2016). Edible Insects of China.

[61] Ma Y.K., Zheng L.J., Sun Z.W., Fu B.R. (2002). Analysis of nutrient components of three species of aquatic beetles. Acta Nutr. Sin..

[62] Guo L.Z., Wang R.L., Liang A.P., Pan S.M., Chen S.H. (2003). Analysis and evaluation of nutritional components of *Hydrous acuminatus*. Entomol. Knowl..

[63] Shi Y.C., Ren Y., Liu H.-B., Gu J., Cao C.Q. (2019). Analysis of Main Nutritional Componets in Hellgrammites and Its Anti-Diuretic Effect. Sci. Technol. Food Ind..

[64] Wang F.B., Liu Y.S. (2011). Analysis and evaluation of resource components of *Neochauliodes sparsus* larvae. Chin. J. Appl. Entomol..

[65] Ramos-Elorduy J., Moreno J.M.P., Prado E.E., Perez M.A., Otero J.L., de Guevara O.L. (1997). Nutritional Value of Edible Insects from the State of Oaxaca, Mexico. J. Food Compos. Anal..

[66] Ghosh S., Namin S., Meyer-Rochow V., Jung C. (2021). Chemical Composition and Nutritional Value of Different Species of *Vespa* Hornets. Foods.

[67] Zhou J., Han D. (2006). Proximate, amino acid and mineral composition of pupae of the silkworm *Antheraea pernyi* in China. J. Food Compos. Anal..

[68] Kiyashko S.I., Imbs A.B., Narita T., Svetashev V.I., Wada E. (2004). Fatty acid composition of aquatic insect larvae *Stictochironomus pictulus* (Diptera: Chironomidae): Evidence of feeding upon methanotrophic bacteria. Comp. Biochem. Physiol. Part B Biochem. Mol. Biol..

[69] Komínková D., Rejmánková E., Grieco J., Achee N. (2012). Fatty acids in anopheline mosquito larvae and their habitats. J. Vector Ecol..

[70] He Z., Sun L., Wang C.Y., Feng Y., Zhao M. (2021). Nutritional composition analysis and evaluation of the two-spotted cricket *Gryllus bimaculatus* (Orthoptera: Gryllidae). Biotic Resources.

[71] Paul A., Frederich M., Megido R.C., Alabi T., Malik P., Uyttenbroeck R., Francis F., Blecker C., Haubruge E., Lognay G. (2017). Insect fatty acids: A comparison of lipids from three Orthopterans and *Tenebrio molitor* L. larvae. J. Asia Pac. Entomol..

[72] Meyer-Rochow V., Gahukar R., Ghosh S., Jung C. (2021). Chemical Composition, Nutrient Quality and Acceptability of Edible Insects Are Affected by Species, Developmental Stage, Gender, Diet, and Processing Method. Foods.

[73] Tomotake H., Katagiri M., Yamato M. (2010). Silkworm Pupae (Bombyx mori) Are New Sources of High Quality Protein and Lipid. J. Nutr. Sci. Vitaminol..

[74] Weng Y.C. (1998). A preliminary study on the nutritional components of *Cybister japonicus* sharp. Fisheries Science & Technology of Guangxi.

[75] Rumpold B.A., Schlüter O.K. (2013). Nutritional composition and safety aspects of edible insects. Mol. Nutr. Food Res..

[76] Wu R.A., Ding Q., Yin L., Chi X., Sun N., Chen R.A.W., Luo L., Ma H., Li Z. (2020). Comparison of the nutritional value of mysore thorn borer (*Anoplophora chinensis*) and mealworm larva (*Tenebrio molitor*): Amino acid, fatty acid, and element profiles. Food Chem..

[77] Yue D.M., Li S.Y., Zhang J., Wei Q., Zhao M.N., Wang L.M. (2017). Analysis on Nutrientional Content and Amino Acid Composition of *Antheraea yamamai* Pupa. Science of Sericulture.

[78] Bhulaidok S., Sihamala O., Shen L. (2010). Nutritional and fatty acid profiles of sun-dried edible black ants (*Polyrhachis vicina* Roger). Maejo Int. J. Sci. Technol..

[79] Lesch V., Bouwman H. (2018). Adult dragonflies are indicators of environmental metallic elements. Chemosphere.

[80] Abidin N.Z., Kormin F., Abidin N.Z., Anuar N.M., Abu Bakar M. (2020). The Potential of Insects as Alternative Sources of Chitin: An Overview on the Chemical Method of Extraction from Various Sources. Int. J. Mol. Sci..

[81] Mohan K., Ganesan A.R., Muralisankar T., Jayakumar R., Sathishkumar P., Uthayakumar V., Chandirasekar R., Revathi N. (2020). Recent insights into the extraction, characterization, and bioactivities of chitin and chitosan from insects. Trends Food Sci. Technol..

[82] Luo Q., Wang Y., Han Q., Ji L., Zhang H., Fei Z., Wang Y. (2019). Comparison of the physicochemical, rheological, and morphologic properties of chitosan from four insects. Carbohydr. Polym..

[83] Kaya M., Baublys V., Can E., Šatkauskienė I., Bitim B., Tubelytė V., Baran T. (2014). Comparison of physicochemical properties of chitins isolated from an insect (*Melolontha melolontha*) and a crustacean species (*Oniscus asellus*). Zoomorphology.

[84] Soon C.Y., Tee Y.B., Tan C.H., Rosnita A.T., Khalina A. (2018). Extraction and physicochemical characterization of chitin and chitosan from Zophobas morio larvae in varying sodium hydroxide concentration. Int. J. Biol. Macromol..

[85] Kaya M., Baran T., Mentes A., Asaroglu M., Sezen G., Tozak K.O. (2014). Extraction and Characterization of α-Chitin and Chitosan from Six Different Aquatic Invertebrates. Food Biophys..

[86] Paulino A., Simionato J.I., Garcia J.C., Nozaki J. (2006). Characterization of chitosan and chitin produced from silkworm crysalides. Carbohydr. Polym..

[87] Ma J., Xin C., Tan C. (2015). Preparation, physicochemical and pharmaceutical characterization of chitosan from *Catharsius molossus* residue. Int. J. Biol. Macromol..

[88] Torres J., Garcia S.R.S., Lara-Villalón M., Ávila G.C.G.M., Mora-Olivo A., Reyes-Soria F.A. (2015). Evaluation of Biochemical Components from *Pterophylla beltrani* (Bolivar & Bolivar) (Orthoptera: Tettigoniidae): A Forest Pest from Northeastern Mexico. Southwest. Entomol..

[89] Ibitoye B.E., Idris L.H., Noor M.M., Meng G.Y., Bakar M.A., Jimoh A.A. (2018). Extraction and physicochemical characterization of chitin and chitosan isolated from House Cricket. Biomed. Mater..

[90] Kaya M., Baran T., Asan-Ozusaglam M., Cakmak Y.S., Tozak K.O., Mol A., Menteş A., Sezen G. (2015). Extraction and characterization of chitin and chitosan with antimicrobial and antioxidant activities from cosmopolitan Orthoptera species (Insecta). Biotechnol. Bioprocess Eng..

[91] Nemtsev S.V., Zueva O.Y., Khismatullin M.R., Albulov A.I., Varlamov V.P. (2004). Isolation of Chitin and Chitosan from Honeybees. Appl. Biochem. Microbiol..

[92] Kim M.-W., Han Y.S., Jo Y.H., Choi M.H., Kang S.H., Kim S.-A., Jung W.-J. (2016). Extraction of chitin and chitosan from housefly, *Musca domestica*, pupa shells. Entomol. Res..

[93] Khayrova A., Lopatin S., Varlamov V. (2019). Black Soldier Fly Hermetia illucens as a Novel Source of Chitin and Chitosan. Int. J. Sci..

[94] Kaya M., Akyuz B., Bulut E., Sargin I., Eroglu F., Tan G. (2016). Chitosan nanofiber production from Drosophila by electrospinning. Int. J. Biol. Macromol..

[95] Kim M.-W., Song Y.-S., Seo D.-J., Han Y.S., Jo Y.H., Noh M.Y., Yang Y.C., Park Y.-K., Kim S.-A., Choi C. (2017). Extraction of Chitin and Chitosan from the Exoskeleton of the Cockroach (*Periplaneta americana* L.). J. Chitin Chitosan.

[96] Nowakowski A.C., Miller A.C., Miller M.E., Xiao H., Wu X. (2021). Potential health benefits of edible insects. Crit. Rev. Food Sci. Nutr..

[97] D’Antonio V., Serafini M., Battista N. (2021). Dietary Modulation of Oxidative Stress From Edible Insects: A Mini-Review. Front. Nutr..

[98] Chakravorty J., Ghosh S., Meyer-Rochow V.B. (2011). Practices of entomophagy and entomotherapy by members of the Nyishi and Galo tribes, two ethnic groups of the state of Arunachal Pradesh (North-East India). J. Ethnobiol. Ethnomed..

[99] Meyer-Rochow V.B. (2017). Therapeutic arthropods and other, largely terrestrial, folk-medicinally important invertebrates: A comparative survey and review. J. Ethnobiol. Ethnomed..

[100] Yang L.F., Tian L.X. (1994). A brief history of aquatic insect research in China. Entomol. Knowl..

[101] Feng Y., Zhao M., He Z., Chen Z., Sun L. (2009). Research and utilization of medicinal insects in China. Entomol. Res..

[102] Zhu L.C. (2012). Applications of Insect Medicine.

[103] Jiang Y.X. (2004). Sympetrum infuscatum as a medicinal dragonfly species in Heilongjiang. Forest By-Prod. Spec. China.

[104] Dossey A.T. (2010). Insects and their chemical weaponry: New potential for drug discovery. Nat. Prod. Rep..

[105] Di Mattia C., Battista N., Sacchetti G., Serafini M. (2019). Antioxidant Activities in vitro of Water and Liposoluble Extracts Obtained by Different Species of Edible Insects and Invertebrates. Front. Nutr..

[106] Zielińska E., Baraniak B., Karaś M. (2017). Antioxidant and Anti-Inflammatory Activities of Hydrolysates and Peptide Fractions Obtained by Enzymatic Hydrolysis of Selected Heat-Treated Edible Insects. Nutrients.

[107] Rinehart K.L., Holt T.G., Fregeau N.L., Keifer P.A., Wilson G.R., Perun T.J., Sakai R., Thompson A.G., Stroh J.G., Shield L.S. (1990). Bioactive Compounds from Aquatic and Terrestrial Sources. J. Nat. Prod..

[108] Feng Y., Chen X.M., Ma Y., He Z. (2006). Experimental study on immunomodulation of white wax scale (*Ericerus pela* Chavannes). For. Res..

[109] Bridges A.R., Owen M.D. (1984). The morphology of the honey bee (*Apis mellifera* L.) venom gland and reservoir. J. Morphol..

[110] Jiang M., Lü S., Zhang Y. (2017). The Potential Organ Involved in Cantharidin Biosynthesis in *Epicauta chinensis* Laporte (Coleoptera: Meloidae). J. Insect Sci..

[111] Mozhui L., Kakati L.N., Meyer-Rochow V.B. (2021). Entomotherapy: A study of medicinal insects of seven ethnic groups in Nagaland, North-East India. J. Ethnobiol. Ethnomed..

[112] Devakul V., Maarse H. (1964). A second compound in the odorous gland liquid of the giant water bug *Lethocerus indicus* (Lep. and Serv.). Anal. Biochem..

[113] Kiatbenjakul P., Intarapichet K.-O., Cadwallader K.R. (2015). Characterization of potent odorants in male giant water bug (*Lethocerus indicus* Lep. and Serv.), an important edible insect of Southeast Asia. Food Chem..

[114] Yang Z.-L., Nour-Eldin H.H., Hänniger S., Reichelt M., Crocoll C., Seitz F., Vogel H., Beran F. (2021). Sugar transporters enable a leaf beetle to accumulate plant defense compounds. Nat. Commun..

[115] Kazana E., Pope T.W., Tibbles L., Bridges M., Pickett J.A., Bones A., Powell G., Rossiter J.T. (2007). The cabbage aphid: A walking mustard oil bomb. Proc. R. Soc. B Boil. Sci..

[116] Kumar P., Pandit S.S., Steppuhn A., Baldwin I.T. (2013). Natural history-driven, plant-mediated RNAi-based study reveals CYP6B46’s role in a nicotine-mediated antipredator herbivore defense. Proc. Natl. Acad. Sci. USA.

[117] Heidari H., Azizi Y., Maleki-Ravasan N., Tahghighi A., Khalaj A., Pourhamzeh M. (2021). Nature’s gifts to medicine: The metabolic effects of extracts from cocoons of *Larinus hedenborgi* (Coleoptera: Curculionidae) and their host plant *Echinops cephalotes* (Asteraceae) in diabetic rats. J. Ethnopharmacol..

[118] Nakane W., Nakamura H., Nakazato T., Kaminaga N., Nakano M., Sakamoto T., Nishiko M., Bono H., Ogiwara I., Kitano Y. (2020). Construction of TUATinsecta database that integrated plant and insect database for screening phytophagous insect metabolic products with medicinal potential. Sci. Rep..

[119] Lidman J., Jonsson M., Berglund M. (2020). The effect of lead (Pb) and zinc (Zn) contamination on aquatic insect community composition and metamorphosis. Sci. Total Environ..

[120] Scheibener S., Conley J.M., Buchwalter D. (2017). Sulfate transport kinetics and toxicity are modulated by sodium in aquatic insects. Aquat. Toxicol..

[121] Neff E., Dharmarajan G. (2020). The direct and indirect effects of copper on vector-borne disease dynamics. Environ. Pollut..

[122] Chaves-Ulloa R., Taylor B.W., Broadley H., Cottingham K.L., Baer N.A., Weathers K.C., Ewing H.A., Chen C.Y. (2016). Dissolved organic carbon modulates mercury concentrations in insect subsidies from streams to terrestrial consumers. Ecol. Appl..

[123] Hare L. (1992). Aquatic Insects and Trace Metals: Bioavailability, Bioaccumulation, and Toxicity. Crit. Rev. Toxicol..

[124] Aydoğan Z., Şişman T., Incekara Ü., Gürol A. (2017). Heavy metal accumulation in some aquatic insects (Coleoptera: Hydrophilidae) and tissues of Chondrostoma regium (Heckel, 1843) relevant to their concentration in water and sediments from Karasu River, Erzurum, Turkey. Environ. Sci. Pollut. Res..

[125] Cain D.J., Luoma S.N., Wallace W.G. (2004). Linking metal bioaccumulation of aquatic insects to their distribution patterns in a mining-impacted river. Environ. Toxicol. Chem..

[126] Eisler R. (2004). Arsenic hazards to humans, plants, and animals from gold mining. Rev. Environ. Contam. Toxicol..

[127] Hall B.D., Rosenberg D.M., Wiens A.P. (1998). Methyl mercury in aquatic insects from an experimental reservoir. Can. J. Fish. Aquat. Sci..

[128] Cristol D.A., Brasso R.L., Condon A.M., Fovargue R.E., Friedman S.L., Hallinger K.K., Monroe A.P., White A.E. (2008). The Movement of Aquatic Mercury Through Terrestrial Food Webs. Science.

[129] Raikow D.F., Walters D.M., Fritz K.M., Mills M.A. (2011). The distance that contaminated aquatic subsidies extend into lake riparian zones. Ecol. Appl..

[130] Kraus J.M., Schmidt T., Walters D.M., Wanty R.B., Zuellig R.E., Wolf R.E. (2014). Cross-ecosystem impacts of stream pollution reduce resource and contaminant flux to riparian food webs. Ecol. Appl..

[131] Wang J.D., Wang C.Y., Zhao M., Feng Y. (2018). Mercury absorption, enrichment and water-land transfer by aquatic insects. Biot. Resour..

[132] Cetinić K.A., Previšić A., Rožman M. (2021). Holo- and hemimetabolism of aquatic insects: Implications for a differential cross-ecosystem flux of metals. Environ. Pollut..

[133] Abu Hassan A., Dieng H., Satho T., Boots M., Al Sariy J.S. (2010). Breeding patterns of the JE vector Culex gelidus and its insect predators in rice cultivation areas of northern peninsular Malaysia. Trop. Biomed..

[134] Marsollier L., Robert R., Aubry J., André J.-P.S., Kouakou H., Legras P., Manceau A.-L., Mahaza C., Carbonnelle B. (2002). Aquatic Insects as a Vector for Mycobacterium ulcerans. Appl. Environ. Microbiol..

[135] Bektaş M., Orhan F., Erman Ö.K., Barış Ö. (2020). Bacterial microbiota on digestive structure of Cybister lateralimarginalis torquatus (Fischer von Waldheim, 1829) (Dytiscidae: Coleoptera). Arch. Microbiol..

[136] Bruus M., Rasmussen J.J., Strandberg M., Strandberg B., Sørensen P.B., Larsen S.E., Kjær C., Lorenz S., Wiberg-Larsen P. (2019). Terrestrial adult stages of freshwater insects are sensitive to insecticides. Chemosphere.

[137] Maloney E., Taillebois E., Gilles N., Morrissey C., Liber K., Servent D., Thany S. (2020). Binding properties to nicotinic acetylcholine receptors can explain differential toxicity of neonicotinoid insecticides in Chironomidae. Aquat. Toxicol..

[138] Jinguji H., Ohtsu K., Ueda T., Goka K. (2018). Effects of short-term, sublethal fipronil and its metabolite on dragonfly feeding activity. PLoS ONE.

[139] Fukuuchi T., Yasuda M., Inazawa K., Ota T., Yamaoka N., Mawatari K.-I., Nakagomi K., Kaneko K. (2013). A Simple HPLC Method for Determining the Purine Content of Beer and Beer-like Alcoholic Beverages. Anal. Sci..

[140] Bednářová M., Borkovcová M., Komprda T. (2013). Purine derivate content and amino acid profile in larval stages of three edible insects. J. Sci. Food Agric..

[141] Kaneko K., Aoyagi Y., Fukuuchi T., Inazawa K., Yamaoka N. (2014). Total Purine and Purine Base Content of Common Foodstuffs for Facilitating Nutritional Therapy for Gout and Hyperuricemia. Biol. Pharm. Bull..

[142] Sabolová M., Kulma M., Kouřimská L. (2020). Sex-dependent differences in purine and uric acid contents of selected edible insects. J. Food Compos. Anal..

[143] He Z., Zhao M., Wang C., Sun L., Jiang Y., Feng Y. (2019). Purine and uric acid contents of common edible insects in Southwest China. J. Insects Food Feed.

[144] Dow J.A.T., Douglas A.E., Chapman R.F., Simpson S.J. (2012). Excretion and salt and water regulation. The Insects: Structure and Function.

[145] Jeong K.Y., Park J.-W. (2020). Insect Allergens on the Dining Table. Curr. Protein Pept. Sci..

[146] De Gier S., Verhoeckx K. (2018). Insect (food) allergy and allergens. Mol. Immunol..

[147] Payne C.L.R., Scarborough P., Rayner M., Nonaka K. (2015). Are edible insects more or less ‘healthy’ than commonly consumed meats? A comparison using two nutrient profiling models developed to combat over- and undernutrition. Eur. J. Clin. Nutr..

[148] Kolakowska A., Olley J.N., Dunstan G.A., Sikorski Z.E., Kolakowska A. (2002). Fish lipids. Chemical and Functional Properties of Food Lipids.

[149] Wang D., Zhai S.-W., Zhang C.-X., Zhang Q., Chen H. (2007). Nutrition value of the Chinese grasshopper *Acrida cinerea* (Thunberg) for broilers. Anim. Feed Sci. Technol..

[150] Twining C.W., Brenna J.T., Hairston N.G., Flecker A.S. (2015). Highly unsaturated fatty acids in nature: What we know and what we need to learn. Oikos.

[151] Honeyfield D.C., Maloney K.O. (2014). Seasonal patterns in stream periphyton fatty acids and community benthic algal composition in six high-quality headwater streams. Hydrobiologia.

[152] Dai Z.H., Liao B.H., Wang Z.H., Wang X.J., Liu Y.X. (1995). A preliminary study on leaching of selenium in the soils of china. J. Environ. Sci..

[153] Lobanov A.V., Fomenko D.E., Zhang Y., Sengupta A., Hatfield D.L., Gladyshev V.N. (2007). Evolutionary dynamics of eukaryotic selenoproteomes: Large selenoproteomes may associate with aquatic life and small with terrestrial life. Genome Biol..

[154] Lobanov A.V., Hatfield D.L., Gladyshev V.N. (2007). Selenoproteinless animals: Selenophosphate synthetase SPS1 functions in a pathway unrelated to selenocysteine biosynthesis. Protein Sci..

[155] Ramos-Elorduy J. (2006). Threatened edible insects in Hidalgo, Mexico and some measures to preserve them. J. Ethnobiol. Ethnomed..

[156] Durst P., Hanboonsong Y. (2015). Small-scale production of edible insects for enhanced food security and rural livelihoods: Experience from Thailand and Lao People’s Democratic Republic. J. Insects Food Feed.

[157] Oonincx D.G.A.B., van der Poel A.F.B. (2010). Effects of diet on the chemical composition of migratory locusts (*Locusta migratoria*). Zoo Biol..

[158] Lancaster J., Downes B.J. (2018). Aquatic versus terrestrial insects: Real or presumed differences in population dynamics?. Insects.

[159] Shaw P.-C., Mark K.-K. (1980). Chironomid farming—A means of recycling farm manure and potentially reducing water pollution in Hong Kong. Aquaculture.

[160] Yokoyama A., Hamaguchi K., Ohtsu K., Ishihara S., Kobara Y., Horio T., Endo S. (2009). A method for mass-rearing caddisfly, *Cheumatopsyche brevilineata* (Iwata) (Trichoptera: Hydropsychidae), as a new test organism for assessing the impact of insecticides on riverine insects. Appl. Entomol. Zool..

[161] Locklin J.L., Huckabee J.S., Gering E.J. (2012). A Method for Rearing Large Quantities of the Damselfly, *Ischnura ramburii* (Odonata: Coenagrionidae), in the Laboratory. Fla. Entomol..

[162] Wang X.L., Wang T.F., Su X.D., Niu D.M. (2019). Study on the techniques of artificial breeding predacious diving beetle. J. Baicheng Norm. Univ..

[163] Wen Y.C., Zhang T.L. (1993). A tentative researche to the large aquatic insect in two classes-predacious diving beetles (Dytiscidae) and giant water burs *Lethocerus indicus* (Lepeletier et Serville). J. Zhanjiang Ocean. Univ..

[164] Van Huis A., Oonincx D.G.A.B. (2017). The environmental sustainability of insects as food and feed. A review. Agron. Sustain. Dev..

[165] Bektas M., Guler O. (2019). Usage of edible aquatic insects for feed rations of poultry animals. Int. J. Sci. Technol. Res..

[166] Henry M., Gasco L., Piccolo G., Fountoulaki E. (2015). Review on the use of insects in the diet of farmed fish: Past and future. Anim. Feed Sci. Technol..

