# Supplementary material for: Edible Aquatic Insects: Diversities, Nutrition, and Safety
Source: Foods. 2021 Dec 6;10(12):3033. doi: 10.3390/foods10123033 (PMC8700862; doi:10.3390/foods10123033)
Supplement: Supplementary file 1 [file foods-10-03033-s001.zip › foods-1463484-supplementary.pdf]

Supplementary Material

Table S1. Full list of named edible aquatic insects. The species without genus name will not put into the table.

Order	Family	Species	Countries	References
Ephemeroptera	Baetidae	*Baetis* sp.	Mexico	[1]
		Cloeon dipterum	Japan	[2,3]
		C. kimminsi	India	[4]
	Caenidae	*Caenis kungu*	Malawi	[5,6]
	Ephemerellidae	*Teleganopsis jinghongensis*	China	[7,8]
		Ephemera danica	India	[4,9]
		Ephemera sp.	Mexico	[1]
		Ephemera sp.	India	[4]
		Sinephemera strigata	Japan	[10]
	Palingeniidae	*Plethogenesia* sp.	Papua New Guinea	[5]
		Povilla adusta	Kenya, Malawi, Tanzania, Uganda	[5,11,12]
Odonata	Aeschnidae	*Aeschna* sp.	Mexico	[1]
		A. mixta	India	[13]
		Aeschna sp.	Thailand	[14]
		Anax guttatus	Indonesia, Laos, Thailand	[15,16]
		An. parthenope	China, Japan	[17]
		Anax sp.	Madagascar, Mexico, Indonesia	[1,6,18]
		Coryphaeschna adnexa	Ecuador	[19]
		Rhionaeschna brevifrons	Ecuador	[19]
		R. multicolor	Mexico, USA	[1,6]
		R. marchali	Ecuador	[19]
		R. peralta	Ecuador	[19]
	Calopterygidae	*Hetaerina* sp.	Venezuela	[20]
		Mnesarete sp.	Venezuela	[20]
		Argia sp.	Venezuela	[20]
		Ceriagrion sp.	Thailand, Laos, Myanmar, Vietnam	[14,21,22]
		Enallagma sp.	India	[9]
		Anotogaster sieboldii	Japan	[17]
		Cordulegaster sp.	India	[23]
	Corduliidae	*Epophthalmia elegans*	China	[24]
		E. vittigera	Thailand	[6,14,21]
		Lauromacromia dubitalis	Venezuela	[20]
		Macromia sp.	Thailand	[17]

Order	Family	Species	Countries	References
	Gomphidae	*Agriogomphus* sp.	Venezuela	[20]
		Davidius nanus	Japan	[17]
		Gomphus cuneatus	China	[7,8]
		G. vulgatissimus	Italy	[25]
		Ictinogomphus rapax	India, China	[4,9,24]
		Progomphus sp.	Venezuela	[20]
		Sinictinogomphus clavatus	China	[26]
		Stylurus sp.	India	[9]
		Zonophora sp.	Venezuela	[20]
	Lestidae	*Lestes praemorsus*	China	[8]
	Libellulidae	*Acisoma panorpoides*	India	[6,23]
		Brachythemis contaminata	India	[4,9]
		Brechmorhoga sp.	Venezuela	[20]
		Cratilla lineata	Indonesia	[16]
		Crocothemis servilia	China, Indonesia, South Korea	[8,16,17,27]
		Crocothermis sp.	Laos, Myanmar, Thailand, Vietnam	[22]
		Dasythemis sp.	Venezuela	[20]
		Diplacodes sp.	India	[9]
		Diplacodes trivialis	India	[4]
		Libellula carolina	India	[13]
		L. pulchella	Thailand	[6]
		Neurothemis fluctuans	India	[13]
		Neurothemis sp.	Indonesia	[6]
		N. ramburii	Indonesia	[16]
		Orthetrum albistylum	China	[28]
		O. glaucum	Indonesia	[16]
		O. japonicum	Japan	[17]
		O. pruinosum	China	[24]
		O. sabina	Indonesia	[16]
		Orthetrum sp.	Malaysia, Sabah	[29]
		O. triangulare	China, Japan	[17,28]
		Pachydiplax sp.	India	[9]
		Pantala flavscens	China, Indonesia	[16,17,30]
		Potamarcha obscura	Indonesia	[16]
		Rhyothemis sp.	Thailand	[6,14,21]
		Sympetrum darwinianum	Japan	[2]

Order	Family	Species	Countries	References
		S. eroticum	Japan	[2]
		S. frequens	Japan	[17]
		S. infuscatum	Japan	[2]
		S. pedemontanum	Japan	[17]
		Sympetrum sp.	India	[9]
		S. speciosum	China	[31]
		S. uniforme	China	[28]
		Tramea transmarina	Indonesia	[15]
		Trithemis arteriosa	D.R.Congo	[32]
		T. aurora	Indonesia	[16]
		Trithemis sp.	Madagascar	[18]
		Urothemis sp.	India	[9]
	Macromiidae	*Macroma* sp.	Thailand	[6]
	Megapodagrionidae	*Oxystigma* sp.	Venezuela	[20]
Plecoptera	Nemouridae	*Nemoura* sp.	India	[4]
	Perlidae	*Kamimuria tibialis*	Japan	[2,3]
		Oyamia gibba	Japan	[17]
		Paragnetina tinctipennis	Japan	[2,3]
		Perla tibialis	Japan	[2]
	Perlodidae	*Isoperla* sp.	USA	[6]
		Perlodes frisonana	Japan	[17]
	Pteronarcyidae	*Pteronarcys californica*	USA	[33]
		P. dorsata	India, USA	[4,34]
		P. princeps	USA	[34]
		P. reticulata	Japan	[17]
Hemiptera	Aphelocheiridae	*Aphelocheirus vitattus*	Japan	[35]
	Belostomatidae	*Abedus dilatatus*	Mexico	[1]
		Ab. ovatus	Mexico	[6]
		Abedus sp.	Mexico	[1,6]
		Belostoma micantulum	Venezuela	[20]
		Belostoma sp.	D.R. Congo, Mexico	[1,6]
		Diplonychus japonicas	Japan	[17]
		D. rusticus	Thailand	[36]
		Diplonychus sp.	Thailand	[21]
		Hydrocyrius punctatus	Madagascar	[18]
		Lethocerus americanus	USA	[6]
		L. cordofanus	Cameroon, Zambezian region	[32,37]
		Le. deyrollei	China, Japan	[2,28]
		Le. indicus	China, India, Laos, Malaysia, Mali,	[4,6,7,9,17,21,22,38]

Order	Family	Species	Countries	References
			Myanmar, Singapore, Sri Lanka; Thailand, Vietnam	
		Lethocerus sp.	Mexico, Republic of Congo	[6,17]
		Le. europdeum	Thailand	[17]
		Limnogeton fieberi	Togo	[39]
		Macrocheraia grandis	India	[23]
		Sphaerodema molestum	Thailand	[6]
		Sp. rustica	China, Indonesia, Thailand	[6,7]
		Sp. molestum	Thailand	[6]
	Corixidae	*Corisella edulis*	Mexico	[1,6]
		Co. texcocana	Mexico	[1,40–42]
		Co. mercenaria	Mexico	[1,6,40,41]
		Corixa esculenta	Egypt	[17]
		Cor. femorale	Mexico	[17]
		Graptocorixa abdominalis	Mexico	[1,40,41]
		Gr. bimaculata	Mexico	[40–42]
		Graptocorixa sp.	Mexico	[43]
		Hesperocorixa distanti	Japan	[17]
		H. laevignata	Mexico	[44]
		Krizousacorixa azteca	Mexio	[1,6,40,41]
		K. femorata	Mexico	[1,6,40,41]
		Micronecta sedula	China	[28]
		Sigara substriata	China	[28]
	Gerridae	*Cylindrostethus scrutator*	Thailand	[14]
		Gerris sp.	India	[6,45]
		Ge. spinole	India	[6]
	Naucoridae	*Ambrysus stali*	Venezuela	[20]
		Am. usingeri	Venezuela	[20]
		Heleocoris naucoroides	Madagascar	[18]
		Limnocorus cf. minutus	Venezuela	[20]
	Nepidae	*Laccotrephes griseus*	Thailand	[6]
		La. japonensis	Japan	[2]
		La. robustus	Indonesia	[15]
		La. ruber	Laos, Thailand	[14,21,22]

Order	Family	Species	Countries	References
		Laccotrephes sp.	Madagascar	[18]
		La. flavovenosa	Japan, South Korea	[17]
		La. maculatus	India	[27]
		Nepa sp.	Madagascar	Decary, 1937
		Nepa sp.	Thailand	[6]
		Ranatra chinensis	Japan, Laos, Thailand	Nonaka, 1999 [2,17]
		Ra. longipes	Thailand	[14,21]
		Ranatra sp.	India	[23]
		Ra. unicolor	Japan	[2]
		Ra. varipes	Thailand	[14,21]
		Sphaerocoris sp.	Malawi	Shaxon et al., 1985
	Notonectidae	*Anisops barbata*	Thailand	[14,21]
		Ani. bouvieri	Thailand	[14,21]
		Ani. nasutus	China	[28]
		Anisops sp.	India, Laos, Myanmar, Vietnam	[22,23]
		Enithares sinica	China	[28]
		Notonecta chinensis	China	[28]
		No. fasciata	Mexico	[44]
		Notonecta sp.	Myanmar	[6]
		Notonecta sp.	Mexico	[1,43]
		No. undulata	Thailand	[21]
		No. unifasciata	Mexico	[1,6]
Megaloptera	Corydalidae	*Acanthacorydalis asiatica*	China	[46]
		Ac. fruhstorferi	China	[46]
		Ac. orientalis	China	[7,8]
		Ac. sinensis	China	[46]
		Ac. unimaculata	China	[46]
		Ac. yunnanensis	China	[46]
		Corydalus armatus	Peru	[47]
		Cs. cornutus	Mexico	[43]
		Corydalus sp.	Colombia, Venezuela	[1,6,20,48]
		Cs. peruvianus	Peru	[47]
		Neoneuromus ignobilis	China	[46]
		Ne. maclachlani	China	[46]
		Parachauliodes japonicas	Japan	[17]
		Protohermes grandis	Japan	[2,49]
		Pr. xanthodes	China	[50]
Coleoptera	Dytiscidae	*Acilius* sp.	China	[51]
		Agabus fulvipennis	China	[51]
		Copelatus sp.	Thailand	[14,21]

Order	Family	Species	Countries	References
		Cybister bengalensis	China	[6,51]
		Cy. brevis	Japan	[2]
		Cy. cinctus	Madagascar	[18]
		Cy. confusus	India	[45]
		Cy. desjardinsii	Madagascar	[18]
		Cy. distinctus	Senegal, Sierra Leone, D.R.Congo	[51]
		Cy. ellipticus	USA	[51]
		Cy. explanatus	Mexico, USA	[6,51,52]
		Cy. fimbriolatus	China, Mexico	[43,51]
		Cy. flavocinctus	China, Mexico	[1,51,52]
		Cy. guerini	China, Indonesia	[6,51]
		Cy. hova	Madagascar	[53]
		Cy. insignis	Gabon	[51]
		Cy. japonicas	China, Japan, North Korea, South Korea	[2,6,51,54–56]
		Cy. lewisianus	China, Laos, Myanmar, Sri Lanka, Thailand, Vietnam	[21,22,38,51]
		Cy. limbatus	China, Laos, Thailand	[6,14,21,22,51,56]
		Cy. occidentalis	Cameroon, China, Mexico	[51,52]
		Cy. operosus	Madagascar	[18]
		Cy. owas	Madagascar	[53]
		Cy. posticus	India	[45]
		Cy. rugosus	Laos, Myanmar, Thailand, Vietnam	[14,21,22]
		Cy. singulatus	China, Japan	[51]
		Cybister sp.	Benin, Cambodia, Malaysia, Sabah, Togo	[29,39,51,57]
		Cybister sp.	Benin, Togo	[57]
		Cybister sp.	Mexico	[52]
		Cybister sp.	Vietnam, Laos, Thailand, Cambodja, Myanmar, Malaysia, Sabah	[6,51]
		Cy. sticticus	China	[17]
		Cy. sugillatus	China, Japan	[6,51]
		Cy. tripunctatus asiaticus	India, Thailand	[14,21]

Order	Family	Species	Countries	References
		Cy. tripunctatus	China, India, Indonesia, Japan, North Korea, South Korea, Sri Lanka, Thailand	[6,15,54,58]
		Cy. ventralis	China	[17]
		Cy. vulneratus	Africa, Madagascar	[18,51]
		Cypris sp.	Thailand	[17,59]
		Dytiscus circumflex	Africa	[51]
		Dy. habilis	China, Japan, Mexico	[51,52]
		Dy. marginalis	China, Japan, Turkey	[6,51,60]
		Dy. marginicollis	Mexico	[52]
		Dytiscus sp.	Cameroon, India	[4,51]
		Dy. validus	China; Japan	[51]
		Eretes sticticus	China, India, Kenya, Laos, Malaysia, Myanmar, Sri Lanka, Thailand; Vietnam	[4,6,14,21,22,51,52]
		Hydraticus rhantoides	Thailand	[14,21]
		Laccophilus apicalis	Mexico	[51]
		Lac. fasciatus	Mexico	[52]
		Lac. pulicarius	Thailand	[14,21]
		Laccophilus sp.	India	[23]
		Laccophilus sp.	Mexico	[61]
		Megadytes giganteus	Mexico	[52]
		M. gigantean	Mexico	[51]
		Megadytus sp.	Mexico	[1,61]
		Platambus guttulus	China	[51]
		Rhantaticus congestus	Thailand	[14,21]
		Rh. atricolor	Mexico	[1,51,61]
		Rh. consimilis	Madagascar, Mexico	[51,52]
		Rh. latus	Madagascar	[51]
		Rh. pulverosus	Japan	[2]
		Rhantus sp.	Mexico	[6,51,61]
		Rh. suturalis	Japan	[2]
		Thermonectus basilaris	Mexico	[52]
		Th. marmoratus	Mexico	[52]
		Thermonetus sp.	Mexico	[61]
	Elmidae	*Austrelmis chilensis*	Chile, Peru	[6,51]
		Au. condimentarius	Chile, Peru, Mexico	[6,51]

Order	Family	Species	Countries	References
	Gyrinidae	*Aulonogyrus strigosus*	Australia	[55]
		Dineutes marginatus	Japan	[2]
		Di. orientalis	Japan	[17]
		Dineutes sp.	Madagascar	[18]
		Gyrinus curtus	Japan	[2]
		Gy. japonicas	Japan	[2]
		Gy. parcus	Mexico	[52]
		Gy. plicatus	Mexico	[52]
		Macrogyrus viridisulcatus	Australia	[62]
		Porrorhynchus marginatus	Japan	[2]
	Haliplidae	*Haliplus punctatus*	Mexico	[52]
		Haliplus sp.	Mexico	[61]
		Peltodytes mexicanus	Mexico	[52]
		Pe. ovalis	Mexico	[52]
		Hololepta guidonis	Mexico	[51]
		Hololepta sp.	Mexico	[6]
	Hydrophilidae	*Berosus* sp.	Mexico	[52]
		Dibolocelus sp.	Mexico	[43,61]
		Hydrobiomorpha spinicollis	Thailand	[21]
		Hy. spinicollis	Thailand	[14]
		Hydrochara sp.	Thailand	[36]
		Hydrophilus acuminatus	China, India, Japan, Mali, North Korea, South Korea	[6,7,17,51,54]
		Hyd. affinis	Japan, Laos	[3,36]
		Hyd. bilineatus	China, Japan, Laos, Myanmar, Thailand, Vietnam	[6,21,22]
		Hyd. cavisternum	China, Japan, Laos, Thailand, Vietnam	[22,51,63]
		Hyd. hastatus	Cambodia, China, Japan, Laos, Myanmar, Thailand, Vietnam	[6,51]
		Hyd. marginatus	Senegal	[51]
		Hyd. olivaceus	India, Sri Thailand	[6,63]
		Hyd. pallidipalpes	China, India	[6,51]
		Hyd. piceus	Turkey	[60]
		Hyd. picicornis	Philippines	[6]
		Hyd. senegalensis	Senegal	[51]

Order	Family	Species	Countries	References
		Hydrophilus sp.	Benin, Togo	[39,43,52]
		Hydrophilus sp.	China	[55]
		Hydrophilus sp.	Thailand, India	[4,6]
		Sternolophus rufipes	Japan, Thailand	[3,14,21]
		Sternolophus sp.	Madagascar	[18]
		Tropisternus collaris	Japan	[51]
		Tr. mexicanus	Mexico, Panama	[51,52]
		Tropisternus sp.	Mexico	[61]
		Tr. sublaevis	Mexico	[51,52]
		Tr. tinctus	Mexico	[6,52]
	Noteridae	*Suphisellus* sp.	Mexico	[51,52]
Diptera	Chaoboridae	*Chaoborus anomalus*	Uganda	[64,65]
		Ch. edulis	Kenya, Malawi, Tanzania, Uganda	[6,17,66]
		Ch. pallidipes	Uganda	[66]
		Chaoborus sp.	East African lakes	[12,66]
		Procladius umbrosus	Uganda	[64]
		Tanypus guttatipennis	Uganda	[64]
	Chironomidae	*Chironomus* sp.	Kenya,Tanzania,Uganda	[12]
	Ephydridae	*Ephydra cinerea*	USA	[6]
		Ep. hians	Mexico, USA	[1,67]
		Ep. macellaria	USA	[6]
		Mosillus tibialis	Mexico	[68]
	Rhagionidae	*Atherix* sp.	Nearctic	[6]
	Simuliidae	*Simulium aureohirtum*	Thailand	[17]
		Si. rubrithorax	Brazil, Venezuela	[6,17,69]
		Simulium sp.	Sri Lanka	[17]
	Stratiomyidae	*Chrysochlorina* sp.	Colombia, Venezuela	[6,48]
	Syrphidae	*Copestylum anna*	Mexico	[1]
		Copestylum haagii	Mexico	[6]
		Eristalis sp.	Mexico	[1]
		Eristalis tenax	Japan	[3,17]
	Tipulidae	*Antocha spinifera*	Japan	[17]
		Holorusia hespera	USA	[6]
		Ho. rubiginosa	N. Am.	[6]
		Tipula derbyi	USA	[6]
		Ti. paludosa	China	[7]
		Ti. quaylii	USA	[6]
		Ti. simplex	USA	[6]

Order	Family	Species	Countries	References
Trichoptera	Calamoceratidae	*Phylloicus* sp.	Venezuela	[20]
	Hydropsychidae	*Cheumatopsyche brevilineata*	Japan	[49]
		Hydropsyche ulmeri	Japan	[17]
		Leptonema sp.	Colombia, Mexico, Venezuela	[6,20,48]
		Macrostemum radiatum	Japan	[17]
	Leptoceridae	*Oecetis disjuncta*	Mexico	[43]
		Triplectides sp.	Venezuela	[20]
	Limnephilidae	*Allophylax* sp.	Japan	[35]
	Odontoceridae	*Marilia* sp.	Venezuela	[20]
	Phryganeidae	*Eubasilissa regina*	Japan	[17]
		Phryganea japonica	Japan	[17]
		Semblis melaleuca	Japan	[17]
	Rhyacophilidae	*Rhyacophila nigrocephala*	Japan	[17]
	Stenopsychidae	*Parastenopsyche sauteri*	Japan	[49]
		Stenopsyche griseipennis	Japan	[6]
		St. marmorata	Japan	[6]
		St. sauteri	Japan	[49]

References

1. Ramos-Elorduy, J.; Pino Moreno, J.M.; Correa, S.C. Edible insects of the state of Mexico and determination of their nutritive values. *An. Inst. Biol. Univ. Nac. Auton. Mex. Ser. Zool.* **1998**, *69*, 65-104.
2. Schmitschek, E. Insekten als nahrung, in brauchtum, kult und kultur. handbuch der zoologie-eine naturgeschichte der stämme des tierreichs. *Handbuch der Zoologie-eine Naturgeschichte der Stamme des Tierreichs* **1968**, *4*, 1-62.
3. Jongema, Y. List of edible insect species of the world. Available online: https://www.wur.nl/en/Research-Results/Chair-groups/Plant-Sciences/Laboratory-of-Entomology/Edible-insects/Worldwide-species-list.htm (accessed on 15 June 2021).
4. Singh, O.; Nabam, S.; Chakravorty, J. Edible insects of Nishi tribe of Arunachal Pradesh. *Hexapoda* **2007**, *14*, 56-60.
5. Grant, P.M. Mayflies as Food. In *Trends in Research in Ephemeroptera and Plecoptera*, Domínguez, E., Ed.; Springer US: Boston, MA, USA, 2001; pp. 107-124.
6. DeFoliart, G.R. *The human use of insects as a food resource: a bibliographic account in progress*; University of Wisconsin: Wisconsin, WI, USA, 2002.
7. Chen, X.M.; Feng, Y. *The edible insects of China*; Beijing, China, 1999; p. 180.
8. Chen, X.M.; Feng, Y.; Chen, Z.Y. Common edible insects and their utilization in China. *Entomol. Res.* **2009**, *39*, 299-303.
9. Chakravorty, J.; Ghosh, S.; Meyer-Rochow, V.B. Practices of entomophagy and entomotherapy by members of the Nyishi and Galo tribes, two ethnic groups of the state of Arunachal Pradesh (North-East India). *J. Ethnobiol. Ethnomed.* **2011**, *7*, 5, doi:10.1186/1746-4269-7-5.
10. Kuwana, S. Important Insect Pests of the Rice Crop in Japan. *Proc. 4th Pac. Sci. Cong.* **1930**, *4*, 209-216.
11. Macadam, C.R.; Stockan, J.A. More than just fish food: ecosystem services provided by freshwater insects. *Ecol. Entomol.* **2015**, *40*, 113-123.
12. Ayieko, M.; Nyambuga, I. *Termites and lake flies in the livelihood of households within the Lake Victoria region: Methods for harvesting and utilization*; Techn. Report for the National Museums of Kenya: Nairobi, Kenya, 2009; pp. 1–11.
13. Ronghang, R.; Ahmed, R. Edible insects and their conservation strategy in Karbi Anglong district of Assam, North East India. *The Bioscan* **2010**, *2*, 515-521.
14. Hanboonsong, Y. Edible insects and associated food habits in Thailand. In Proceedings of the Forest insects as food: humans bite back, Proceedings of a workshop on Asia-Pacific resources and their potential for development, Chiang Mai, Thailand, 2010; pp. 173-182.
15. van der Meer Mohr, J.C. Insects eaten by the Karo-Batak people (a contribution to entomo-bromatology). *Entomologische Berichten* **1965**, *25*, 101-107.
16. Césard, N. Des libellules dans l'assiette: les insectes consommés à Bali. *Insectes* **2006**, *140*, 3-6.
17. Mitsuhashi, J. *Edible insects of the world*; CRC press: Boca Raton, FL, USA, 2016; p. 296.

18. Randrianandrasana, M.; Berenbaum, M.R. Edible non-crustacean arthropods in rural communities of Madagascar. *J. Ethnobiol.* **2015**, *35*, 354-383.
19. Onore, G. Edible insects in Ecuador. In *Ecological Implications of Minilivestock: Potential of Insects, Rodents, Frogs, and Snails*, Paoletti, M.G., Ed.; Science Publishers Inc.: Enfield, NH, USA, 2005; pp. 343-352.
20. Araújo, Y.; Beserra, P. Diversidad en invertebrados consumidos por las etnias Yanomami y Yekuana del Alto Orinico, Venezuela. *Interciencia* **2007**, *32*, 318-323.
21. Hanboonsong, Y.; Rattanapan, A.; Utsunomiya, Y.; Masumoto, K. Edible insects and insect-eating habits in northeastern Thailand. *Elytra* **2000**, *28*, 355-364.
22. Yhoung-Aree, J.; Viwatpanich, K. Edible Insects in the Laos PDR, Myanmar, Thailand, and Vietnam. In *Ecological implications of minilivestock: potential of insects, rodents, frogs and snails*, Paoletti, M.G., Ed.; Science Publishers Inc.: Enfield, NH, USA, 2005; pp. 415-440.
23. Meyer-Rochow, V. Traditional food insects and spiders in several ethnic groups of Northeast India, Papua New Guinea, Australia, and New Zealand. In *Ecological implications of minilivestock: potential of insects, rodents, frogs, and snails*, Paoletti, M.G., Ed.; Science Publishers Inc. : Enfield, NH, USA, 2004; pp. 385-409.
24. Jiang, Y.Y.; Zhao, M.; He, Z.; Wang, C.Y.; Sun, L.; Feng, Y. Nutrition composition and evaluation of six edible dragonfly naiads. *Biotic. Resour.* **2017**, *39*, 352-359, doi:10.14188/j.ajsh.2017.05.007.
25. Boano, G.; Rolando, A. Aggressive interactions and demographic parameters in Libellula fulva (Odonata, Libellulidae). *Ital. J. Zool.* **2003**, *70*, 159-166.
26. Li, S.Y. Preliminary report on national edible insect resources in Pu'er Yunnan. *Southwest China J. Agr. Sci.* **2011**, *3*, 1195-1202.
27. Shantibala, T.; Lokeshwari, R.K.; Debaraj, H. Nutritional and antinutritional composition of the five species of aquatic edible insects consumed in Manipur, India. *J. Insect Sci.* **2014**, *14*, 14, doi:10.1093/jis/14.1.14.
28. Hu, P.; Zha, L.S. Records of Chinese Edible Insects. *J. Anhui Agr. Sci.* **2010**, *10*, 114-118.
29. Chung, A.Y.C.; Khen, C.V.; Unchi, S.; Binti, M. Edible insects and entomophagy in Sabah, Malaysia. *Malay. Nat. J.* **2002**, *56*, 131-144.
30. Lukiwati, D.R. Teak caterpillars and other edible insects in Java. In Proceedings of the Forest insects as food: humans bite back, Proceedings of a workshop on Asia-Pacific resources and their potential for development, Chiang Mai, Thailand, 2010; pp. 99-103.
31. Wang, C.Y.; Zhao, M.; Wang, J.D.; Jiang, Y.Y.; He, Z.; Feng, Y. Molecular identification of a new species of edible dragonfly. *Biotic. Resour.* **2018**, *40*, 164-169, doi:10.14188/j.ajsh.2018.02.011.
32. Malaisse, F. Se nourrir en forêt claire africaine. Approche écologique et nutritionnelle. *Nature Sciences Société* **1999**, *3*, 88.
33. Sutton, M.Q. The California salmon fly as a food source in northeastern California. *J. California Great Basin Anthropol.* **1985**, *7*, 176-182.
34. Sutton, M.Q. *Insects as food: aboriginal entomophagy in the Great Basin*; Ballena Press: Menlo Park, CA, USA., 1988; pp. 1-155.
35. Torii, C. A note on Zazamushi, a speciality of Tenryū River in Ina. *Shin Konchû* **1957**, *10*, 26–29.
36. Wongsiri, S.; Chen, P.P. Effects of agricultural development on honey bees in Thailand. *Bee World (United Kingdom)* **1995**, *76*, 3-5.
37. De Colombel, V. Les insects chez dix populations de langue Tchadique (Cameroun). In *Les insects dans la tradition orale*, Motte-Florac, E., Thomas, J.M.C., Eds.; Peeters: Leuven, Belgium, 2003; pp. 45-62.
38. Nandasena, M.R.M.P.; Disanayake, D.M.S.K.; Weeratunga, L. Sri Lanka as a potential gene pool of edible insects. In Proceedings of the Forest insects as food: humans bite back, Proceedings of a workshop on Asia-Pacific resources and their potential for development, Chiang Mai, Thailand, 2010; pp. 161-164.
39. Tchibozo, S. Les insectes comestibles d'Afrique de L'Ouest et Centrale sur Internet. Available online: http://gbif.africamuseum.be/lincaocnet (accessed on 22 April 2021).
40. Ramos-Elorduy, J. Anthropo-entomophagy: Cultures, evolution and sustainability. *Entomol. Res.* **2009**, *39*, 271-288, doi:10.1111/j.1748-5967.2009.00238.x.
41. Ramos-Elorduy, J. Threatened edible insects in Hidalgo, Mexico and some measures to preserve them. *J. Ethnobiol. Ethnomed.* **2006**, *2*, 51, doi:https://doi.org/10.1186/1746-4269-2-51.
42. Cerritos, R. Insects as food: An ecological, social and economical approach. *CAB Reviews: Perspectives in Agriculture, Veterinary Science, Nutrition and Natural Resources* **2009**, *4*, 1-10, doi:10.1079/PAVSNNR20094027.
43. Ramos-Elorduy, J.; Pino Moreno, J.M. Edible insects of chiapas, Mexico. *Ecol. Food Nutr.* **2002**, *41*, 271-299.
44. Ramos-Elorduy, J.; Pino Moreno, J.M.; Escamilla Prado, E.; Alvarado Perez, M.; Lagunez Otero, J.; Ladron de Guevara, O. Nutritional Value of Edible Insects from the State of Oaxaca, Mexico. *J. Food Compost. Anal.* **1997**, *10*, 142-157, doi:10.1006/jfca.1997.0530.
45. Sangma, R.H.C.; Pal, R.; Singh, D.R. Edible Insects of Northeast India. In *Bioprospecting of Indigenous Bioresources of North-East India*, Purkayastha, J., Ed.; Springer: Singapore, 2016; pp. 253-267.
46. Cao, C.Q. Situation of developing and utilizing sand crawling worm in China and species definition. *Hubei Agr. Sci.* **2014**, *53*, 5061-5064.
47. Paoletti, M.G.; Dufour, D.L. Edible Invertebrates among Amazonian Indians: A Critical Review of Disappearing Knowledge. In *Ecological Implications of Minilivestock: Potential of Insects, Rodents, Frogs and Sails*, Paoletti, M.G., Ed.; Science Publishers Inc.: Enfield, NH, USA, 2005; pp. 293–342.
48. Ruddle, K. The human use of insects: examples from the Yukpa. *Biotropica* **1973**, *5*, 94-101.
49. Mitsuhashi, J. Insects as traditional foods in Japan. *Ecol. Food Nutr.* **1997**, *36*, 187-199.

50. Chen, S.Z.; Yi, L.L.; Ren, W.Y.; Huang, C.J.; Cao, C.Q. Oviposition habits and biological characteristics of Protohermes xanthodes. *Jiangsu Agr. Sci.* **2012**, *40*, 366-368.
51. Ramos-Elorduy, J.; Pino Moreno, J.M.; Martínez Camacho, V.H. Edible aquatic Coleoptera of the world with an emphasis on Mexico. *J. Ethnobiol. Ethnomed.* **2009**, *5*, 11, doi:10.1186/1746-4269-5-11.
52. Ramos-Elorduy, J.; Pino Moreno, J.M. Los coleoptera comestibles de México. *Anales del Instituto de Biología. Serie Zoología* **2004**, *75*, 149-183.
53. Decary, R. L'entomophagie chez les indigènes de Madagascar. *Bull. Soc. Entomol. Fr.* **1937**, *42*, 168-171.
54. Meyer-Rochow, V.B. Ethno-entomological observations from North Korea (officially known as the "Democratic People's Republic of Korea"). *J. Ethnobiol. Ethnomed.* **2013**, *9*, 1-8.
55. Jäch, M.A. Fried water beetles Cantonese style. *Am. Entomol.* **2003**, *49*, 34-37.
56. Feng, Y.; Chen, X.M.; Zhao, M. *Edible Insects of China*; Science press: Beijing, China, 2016; pp. 1-313.
57. Riggi, L.; Veronesi, M.; Verspoor, R.; MacFarlane, C. *Exploring Entomophagy in Northern Benin - Practices, Perceptions and Possibilities*; Bugs for life: London, UK, 2013; pp. 1-40.
58. Pemberton, R.W. Contemporary use of insects and other arthropods in traditional Korean medicine (hanbang) in South Korea and elsewhere. In *Ecological Implications of Minilivestock: Potential of Insects, Rodents, Frogs and Sails*, Paoletti, M.G., Ed.; Science Publishers Inc.: Enfield, NH, USA, 2005; pp. 459-474.
59. Lumsa-ad, C. Study on the species and the nutrition values of edible insects in upper southern Thailand. *Kaen Kaset* **2001**, *29*, 45–49.
60. İncekara, Ü.; Türkez, H. The genotoxic effects of some edible insects on human whole blood cultures. *Munis Entomol. Zool.* **2009**, *4*, 531-535.
61. Ramos-Elorduy, J. La etnoentomología en la alimentación, la medicina y el reciclaje. In *Biodiversidad, Taxonomía y Biogeografía de Artrópodos de México: hacia una síntesis de su conocimiento*, Llorente, J.B., Morrone, J., Yanez, O.O., Vargas, I.F., Eds.; National University Press: Mexico City, Mexico, 2004; pp. 329-413.
62. Welch, D.M. Aboriginal culture. Available online: https://www.aboriginalculture.com.au/ (accessed on 6 April 2021).
63. Utsunomiya, Y.; Masumoto, K. Edible beetles (Coleoptera) from northern Thailand. *Elytra* **1999**, *27*, 191-198.
64. Bergeron, D.; Bushway, R.J.; Roberts, F.L.; Kornfield, I.; Okedi, J.; Bushway, A.A. The nutrient composition of an insect flour sample from Lake Victoria, Uganda. *J. Food Comp. Anal.* **1988**, *1*, 371-377, doi:https://doi.org/10.1016/0889-1575(88)90038-5.
65. MacDonald, W.W. Observations on the biology of chaoborids and chironomids in Lake Victoria and on the feeding habits of the elephant-snout fish'(Mormyrus kannume Forsk.). *J. Anim. Ecol.* **1956**, *25*, 36-53.
66. Bergier, E. *Peuples entomophages et insectes comestibles: étude sur les moeurs de l'homme et de l'insecte*; Imprimerie Rulliere Freres: Avignon, France, 1986.
67. Delong, D.M. Man in a world of insects. *The Ohio J. Sci.* **1960**, *60*, 193.
68. Ramos-Elorduy, J.; Pino Moreno, J.M.; Márquez, C.; Rincón, F.; Alvarado, M.; Escamilla, E.; Bourges, H. Protein content of some edible insects in Mexico. *J. Ethnobiol.* **1984**, *4*, 61-72.
69. Costa Neto, E.M.; Ramos-Elorduy, J. Los insectos comestibles de Brasil: etnicidad, diversidad e importancia en la alimentación. *Boletín Sociedad Entomológica Aragonesa* **2006**, *38*, 423-442.
